# The characteristics of excitatory lineage differentiation and the developmental conservation in *Reeler* neocortex

**DOI:** 10.1111/cpr.13587

**Published:** 2023-12-12

**Authors:** Huan‐Huan Deng, Shi‐Yuan Tong, Dan Shen, Shu‐Qing Zhang, Yinghui Fu

**Affiliations:** ^1^ Jing'an District Central Hospital of Shanghai, State Key Laboratory of Medical Neurobiology, MOE Frontiers Center for Brain Science, Institutes of Brain Science Fudan University Shanghai China

## Abstract

The majority of neocortical projection neurons are generated indirectly from radial glial cells (RGCs) mediated by intermediate progenitor cells (IPCs) in mice. IPCs are thought to be a great breakthrough in the evolutionary expansion of the mammalian neocortex. However, the precise ratio of neuron production from IPCs and characteristics of RGC differentiation process are still unclear. Our study revealed that direct neurogenesis was seldom observed and increased slightly at late embryonic stage. Besides, we conducted retrovirus sparse labelling combined carboxyfluorescein diacetate succinimide ester (CFSE) and *Tbr2‐CreER* strain to reconstruct individual lineage tree in situ. The lineage trees simulated the output of RGCs at per round of division in sequence with high temporal, spatial and cellular resolution at P7. We then demonstrated that only 1.90% of neurons emanated from RGCs directly in mouse cerebral neocortex and 79.33% of RGCs contributed to the whole clones through IPCs. The contribution of indirect neurogenesis was underestimated previously because approximately a quarter of IPC‐derived neurons underwent apoptosis. Here, we also showed that abundant IPCs from first‐generation underwent self‐renewing division and generated four neurons ultimately. We confirmed that the intermediate proliferative progenitors expressed higher *Cux2* characteristically at early embryonic stage. Finally, we validated that the characteristics of neurogenetic process in lineages and developmental fate of neurons were conserved in *Reeler* mice. This study contributes to further understanding of neurogenesis in neocortical development.

## Graphical abstract

1. The differentiation process in lineage is illustrated by sparse labelling.
2. 93.03% of neuronal production are mediated by intermediate progenitor cells.
3. Half of intermediate progenitor cells at first generation are proliferative and contribute to part of superficial neurons.
4. The intermediate proliferative progenitor cells express high level of *Cux2*.
5. The differentiation process of lineage is conserved in *Reeler* mice.

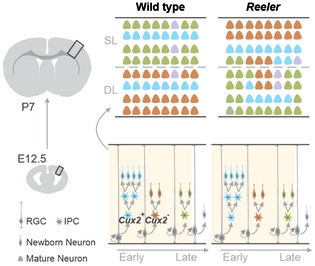

## 1 INTRODUCTION

Neocortex, the largest part of the mammalian cerebral cortex, is the anatomical basis of higher cognitive function. It consists of 85% excitatory projection neurons (PNs) and 15% inhibitory interneurons (INs).^1^ The vast majority of neocortical excitatory neurons originate from radial glial cells (RGCs) located in the proliferative area adjacent to the ventricular zone (VZ).^2^, ^3^, ^4^ Generally, RGCs divide symmetrically to expand the progenitor cell pool at the beginning of neurogenesis, as neurogenesis proceeds, RGCs divide asymmetrically to generate neurons directly or indirectly.^5^ Indirect neurogenesis is mediated by intermediate progenitor cells (IPCs) that undergo symmetrical divisions predominantly in the subventricular zone (SVZ) to produce two (or more) neurons.^6^, ^7^ Certainly, RGCs also generate a few basal radial glial cells (bRGCs) which divide in SVZ.^8^, ^9^, ^10^ Cortical IPCs are a unique cell type in vertebrate neurogenesis, as a substantial strategy to increase the neuron production during neocortex expansion.^11^, ^12^, ^13^, ^14^ Analysis of the RGCs division patterns in rats has documented that the progenies in asymmetric division are more neurons generated directly by RGCs.^7^ However, Kowalczyk et al. proposed that IPCs contributed probably the majority of PNs (more than 80%) to neocortex.^15^ This conjecture has been put forward in a number of subsequent articles that IPCs might be the transitional state between RGCs and cortical glutamate neurons.^6^, ^16^, ^17^, ^18^ So far, the precise ratio of neuron production from IPCs throughout neurogenesis in mice has never been elaborated experimentally and comprehensively.

For a long time, IPCs were considered that they principally participated in the generation of abundant superficial neurons at late developmental stage.^19^, ^20^ However, accumulating evidence suggests that IPCs participate in each laminar formation throughout corticogenesis.^18^, ^21^, ^22^ Moreover, the early IPCs generate neurons in both deep and superficial layers, and late IPCs only generate neurons in the superficial layers.^21^ Generally, neuron production follows the basic neurogenesis rule defined as ‘inside‐out’ pattern,^23^ that is, early‐born neurons reside in deeper cortical plate (CP) while late‐born neurons migrate into superficial layers across the neurons in deep layers. Therefore, this cohort of superficial neurons originated from early IPCs do not follow the ‘inside‐out’ rule. This phenomenon leads to two hypotheses: (1) the early IPCs are committed to maintain quiescent until late neurogenesis; (2) the early IPCs go through another cell cycle to amplify IPCs ahead of the last neurogenetic division.^21^, ^22^ Although the quiescent status of IPCs has never been reported before in neurogenesis, the existence of intermediate proliferative progenitor cells (IPPs) has been observed in murine.^7^, ^24^ Actually, IPPs are common in humans and tree shrews because of their higher proliferative capacity of IPCs.^25^, ^26^, ^27^ However, the characteristics of IPPs are still poorly understood. Interestingly, a previous study elaborated that a subset of neuronal progenitors in the SVZ specifically generated superficial PNs,^20^ and they shared similar identities including origin and output when compared with IPPs. Furthermore, this cohort of progenitor cells express a transcription factors cut‐like 2 (*Cux2*), which is also expressed in their progenies in CP.^20^, ^28^ Therefore, there may be some relationships between *Cux2*‐expressing progenitors and IPPs.

Indeed, the neurogenic fashion not only directly influences the neuronal number during neocortical expansion, but also affects the establishment of neural circuits and the assembly of functional columns. Understanding the stepwise neurogenic process in a lineage could effectively facilitate to elucidate the neocortical development. Previous studies on lineage have elucidated certain neurogenesis patterns but still lack comprehensive cognition. Division of individual RGC was captured by time‐lapse imaging, but it was difficult to achieve long‐term tracking of a complete lineage in vitro.^6^, ^29^, ^30^ Genetic manipulation was used to track the fate of lineages expressing specific transcription factors (like Fezf2), but the limitation of this method was that it cannot present the differentiation process of RGCs in the lineages.^31^ In addition, sparse labelling via mosaic analysis with the double markers (MADM) systems or retrovirus illustrated the spatial location or identity of progenies in a lineage. However, the differentiation process and generational sequence of these neurons with heterogeneous layer or identity cannot be depicted in these studies.^32^, ^33^ Alternatively, single‐cell RNA sequencing (scRNA‐seq) technology is the most promising method to elucidate the development of neocortical lineage. scRNA‐seq was employed with barcodes to trace lineages and their progenies, but the differentiation process and layer information were blurred.^34^, ^35^, ^36^, ^37^, ^38^, ^39^ Therefore, we need to develop a suitable strategy to comprehensively understand the stepwise neurogenic process in a lineage.

In our experiment, we addressed the proportion of IPCs contribution to neurogenesis in meso and clone scales. We developed a novel method of sparse lineage tracing and reconstructed 237 lineages with high temporal and cellular resolution. Then, we depicted the neurogenesis process of individual lineage and summarized their characteristics. In addition, plentiful IPPs were identified in the lineages and they expressed high level of *Cux2* characteristically compared to non‐proliferative IPCs. Finally, in order to investigate whether the process of neocortical lineage differentiation and their characteristics are inherently determined by RGCs, we introduced *Reeler* mouse,^40^ in which Reelin expression is abnormal. Reelin‐Dab1 signalling pathway controls the neuron migration and may function in the nucleus.^41^, ^42^ Thus, *Reeler* mouse provides an appropriate model for investigating the conservation of RGC differentiation in aberrant neocortex. We also conducted sparse lineage tracing and identity verification in *Reeler* mouse. The results revealed that the characteristics of neurogenetic process and neuronal fate were conserved in *Reeler* mouse.

## 2 MATERIALS AND METHODS

### 2.1 Animals

*Tbr2‐CreER* ^*T2*^ mice were kindly provided by Miao He, *Reeler* mice were purchased from Shanghai Model Organisms Center. The *tdTomato* reporter mice (*Ai14‐tdTomato*) and *EGFP* reporter mice (*Ai140‐EGFP*) were purchased from the Jackson Laboratory. CD1 mice used in this study were purchased from B&K Universal Group Limited. Animals were housed under standard conditions (12‐h light/dark cycle). The vaginal plug date was observed at E0.5 (embryonic day) and the birth date was designated as P0.5 (postnatal day). All animal procedures were performed in accordance with the guidelines for animal research and their use by Fudan University.

### 2.2 Tamoxifen and bromodeoxyuridine/idoxuridine administration

For *Tbr2‐CreER* ^*T2*^ mice, pregnant dams were intraperitoneally administered tamoxifen (TM; T5648, Sigma‐Aldrich; 10 mg/kg) and progesterone (P3972, Sigma‐Aldrich; 5 mg/kg) dissolved in corn oil. Embryos were acquired at E18.5 through caesarean section, and they were fostered by other synchronous lactating females. Brains were collected at P7 for further analysis. Fresh bromodeoxyuridine (BrdU, B5002, Sigma‐Aldrich) or idoxuridine (IdU, 1336001, USP) solution (concentration 50 mg/kg) was injected intraperitoneally into pregnant females at the indicated embryonic ages.

### 2.3 Immunohistochemistry

Embryonic brains were perfused with phosphate buffer saline (PBS, pH 7.4) and fixed in 4% paraformaldehyde (PFA) overnight at 4°C. Coronal sections 30–80 μm in thickness were cut using a vibrating microtome (Leica, VT100S), and sections 10 μm in thickness were embedded in Optimal Cutting Temperature (OCT) medium (22110617, Epredia™) and cut on a cryostat (RWD Life Science). Antigen retrieval was performed by incubating the sections in citrate buffer solution (P0083, beyotime) and boiling twice, sections were rinsed three times in PBS after cooled down to room temperature. Then sections were blocked in 10% donkey serum containing 0.5% Triton X‐100 for 2 h at room temperature or overnight at 4°C. Primary antibody incubation was subsequently performed for 48 h at 4°C, the primary antibodies used were chicken anti‐GFP (Aves lab, GFP‐1020), Sheep anti‐Tbr2 (R&D system, AF6166), Rat anti‐Tbr2 (Thermo Fisher Scientific, 14487582, 1:1000), Mouse anti‐IdU (Sigma‐Aldrich, SAB3701448), Rat anti‐BrdU (Abcam, ab6326), Rabbit anti‐Pax6 (MBL, PD022), Sheep anti‐Pax6 (R&D system, AF8150), Rat anti‐CD31 (BD Pharmingen, 550274), Mouse anti‐FITC (Abcam, ab112511), Rabbit anti‐Ki67 (Abcam, ab15580), Rabbit anti‐RFP (Rockland, 600,401,379), Mouse anti‐Satb2 (Abcam, ab51502), Rat anti‐Ctip2 (Abcam, ab18465) and Rabbit anti‐Fog2 (Abcam, custom). Sections were then washed three times in PBS containing 0.1% Triton X‐100 (PBST) and followed by secondary antibody incubation at room temperature for 2 h. The secondary antibodies included Donkey anti‐Rabbit 405 (Abcam, ab175651), Donkey anti‐Rat 405 (Abcam, ab175670), Goat anti‐Mouse 405 (Invitrogen, A31553), Donkey anti‐Mouse 488 (Invitrogen, A21202), Donkey anti‐Rat 488 (Invitrogen, A21208), Donkey anti‐Chicken 488 (Jackson, 703005155), Donkey anti‐Sheep 568 (Invitrogen, A21099), Donkey anti‐Rabbit 568 (Invitrogen, A10042), Donkey anti‐Mouse 555 (Invitrogen, A31570), Goat anti‐Rat 594 (Invitrogen, A11007), Donkey anti‐Rabbit 546 (Invitrogen, A10040), Donkey anti‐Mouse 647 (Invitrogen, A31571), Donkey anti‐Rat 647 (Abcam, ab150155) and Donkey anti‐Rabbit 647 (Invitrogen, A31573). Sections were washed three times with PBS and were dry‐mounted on slides using Fluoromount (F4680, Sigma‐Aldrich). Nuclei were counterstained with Hoechst for 15 min (14,533, Sigma‐Aldrich). For IdU and BrdU antibody staining, sections were denatured before blocking by incubating them in 2 N HCl at room temperature for 2 h, and neutralized with sodium borate buffer followed by washing three times in PBS before blocking.

### 2.4 RNA scope

Mouse brain (E12.5 and E13.5) infected by IdU and BrdU were perfused with PBS and fixed in 4% PFA which were treated by diethyl pyrocarbonate. After dehydration with 30% sucrose in RNase free PBS overnight at 4°C, the brains were embedded in OCT compound, then they were cut coronally at 10 μm thickness with a cryostat (RWD Life Science) and mounted onto SuperFrost Plus Microscope slides (Thermo Fisher Scientific). The frozen brain sections were manipulated using the *Cux2* probe (Mm‐Cux2‐C2, 469551‐C2; diluted with Probe diluent, 300041, Advanced Cell Diagnostics) and RNA‐scope Multiplex Fluorescent Reagent Kit (323100, Advanced Cell Diagnostics) according to the provided protocols. The brain sections were treated by hydrogen peroxide for 10 min at room temperature and washed in distilled water. Subsequently, they were boiled with target retrieval reagent for 2 min at 100°C. After washed with distilled water and 100% ethanol incubation for 3 min, they were incubated with protease IV for 30 min at 40°C. After washed with PBS, *Cux2* probe was added to sections and incubated for 2 h at 40°C. Continuously incubated with AMP1 (30 min, 40°C), AMP2 (30 min, 40°C), AMP3 (15 min, 40°C), HRP‐C2 (15min, 40°C), Opal 520 (30 min, 40°C) and HRP blocker (15 min, 40°C), sections were washed with the washing buffer between two procedures. Ultimately, common immunohistochemistry was carried out as usual.

### 2.5 In utero intraventricular injection

Timed pregnant CD1 mice were anaesthetised by isoflurane. Then, we placed them on a warm operating table and exposed the uterine horns with a small abdominal incision. Retrovirus (PUX‐EGFP, PUX‐Cre, OBiO) mixed with 1% fast green (2.5 mg/mL, Sigma‐Aldrich) or CFSE (C34554, C34564, Thermo Fisher Scientific) was injected into the embryonic cerebral ventricle through a bevelled, calibrated glass micropipette (Drummond Scientific). CFSE was transfused 15 min after retrovirus injection when we traced lineage sparsely. After injection, the uterus was placed back into the abdominal cavity and the peritoneal cavity was infused with 10 mL warm PBS containing Penicillin–Streptomycin solution (15140122, Gibco), the wound was surgically sutured. Then, the animal was placed into a thermostat until it fully recovered.

### 2.6 Confocal time‐lapse imaging

Sixteen hours after retroviral injection, embryonic brains were perfused and sectioned coronally in glacial artificial cerebrospinal fluid  enriched with oxygen. Slices were transferred onto slice culture inserts (Millicell) in culture well plates (Gibco) with culture medium containing 67% dulbecco's modified eagle medium, 25% Hank's balanced salt solution, 5% fetal bovine serum, 1% N2 and 2% Pen/Strep (Gibco). Green fluorescent protein (GFP)‐labelled progenitor cells near the ventricular surface were imaged on an inverted Olympus confocal microscope (IXplore SpinSR). Projection images were acquired from 200 μm Z‐stacks with time series tracking of individual GFP^+^ cell, images were kept at least 48 h with 30‐min interval at 1024 × 1024 pixels resolution. During the whole procedure, slices were maintained in a humidified incubator at 37°C, 5% CO_2_. Finally, montages were assembled, time‐lapse sequences were arranged, and images were adjusted to improve contrast/brightness using Imaris. Line drawings were completed using Adobe Premiere.

### 2.7 Image acquisition and processing

Sections were imaged on an inverted confocal microscope (Fluoview FV3000, Olympus) and projection images with maximum intensity were acquired. Images were analysed using Imaris and adjusted for contrast and brightness in Adobe Photoshop. Data were collected from at least three brains per timepoint and presented as mean ± SEM. For 3D reconstruction of lineages, whole brain sections photographed by Olympus VS120 were reconstructed for clones spanning several 80‐μm adjacent sections using Neurolucida. Neurons and glia were distinguished based on their morphology, and cortical areas were identified using the Allen Brain Atlas. The intensities of CFSE in determinate lineage were captured using Live Cell Imaging System (UltraVIEW VOX, Andor and SpinsR, Olympus).

### 2.8 Statistical analysis

Schematic dendrogram and Alluvial plot were performed using python. All data were reported as mean ± SEM. Unpaired *t*‐test or Mann–Whitney test was performed to examine significant difference between two groups. Statistical significance between more than two groups was determined using two‐way analysis of variance (ANOVA).

## 3 RESULTS

### 3.1 Neocortical development is predominately driven by indirect neurogenesis

**FIGURE 1:**
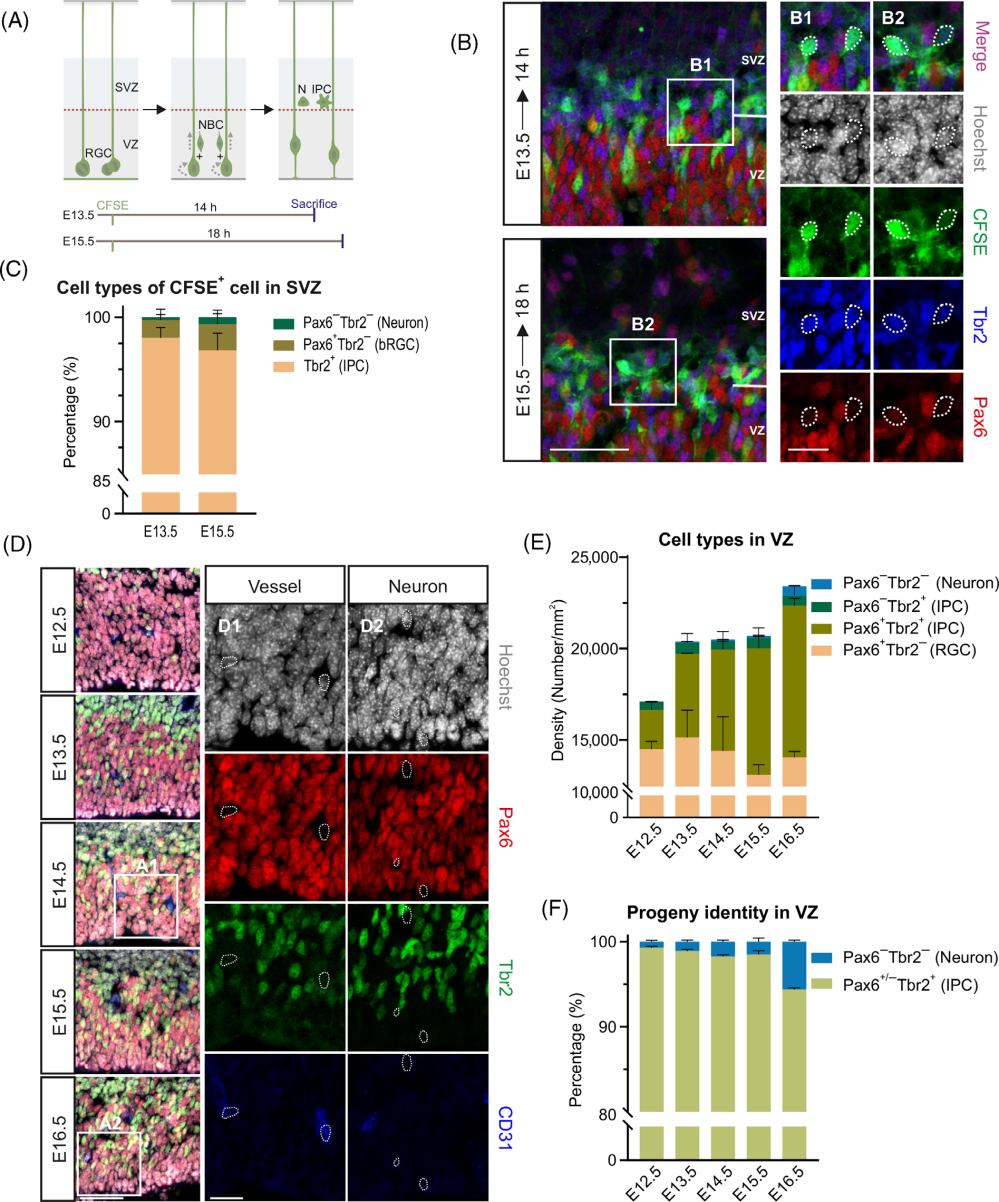
Indirect neurogenesis is the main source of neocortical glutamatergic neurons. (A–C) Labelling the cells which new‐arriving at SVZ generated from RGCs by CFSE, cell types are distinguished by the transcription factor expression while they arriving at SVZ. (A) Schematic drawing of NBCs from RGCs labelled by CFSE at E13.5 and E15.5. We test the identity of NBCs including IPCs and Neurons (N) when the NBCs arrive at SVZ, and the time windows are 14 h at E13.5 and 18 h at E15.5, respectively. (B) Immunostaining of CFSE stained with Tbr2 (blue) and Pax6 (red). Nuclei are labelled with Hoechst (white). High‐magnification images are shown on the right (B1 and B2). Scale bar: 50 μm (left) and 20 μm (right). (C) Percentage of the cell types in CFSE^+^ cells in SVZ. *n* = 3 for wild‐type brains. (D–F) Cell types are distinguished by the expression of transcription factor in VZ. (D) Immunofluorescent staining of embryonic neocortex from E12.5 to E16.5. Pax6 (red), Tbr2 (green) and CD31 (blood vessel endothelium, blue) triple immunohistochemistry, combined with Hoechst (white) staining distinguish four types of cells: Hoechst^+^Pax6^−^Tbr2^−^CD31^+^ (vascular endothelium cell, as shown in D1), Hoechst^+^Pax6^−^Tbr2^−^CD31^−^ (neuron, as shown in D2), Hoechst^+^Pax6^+^Tbr2^−^CD31^−^ (RGC), Hoechst^+^Pax6^+/−^Tbr2^+^CD31^−^ (IPC). Scale bar: 50 μm (left) and 20 μm (right). (E) Quantification of the density of RGC, IPC and Neuron in VZ. *n* = 3 for wild‐type brains. (F) Percentage of the IPCs and neurons in VZ. CFSE, carboxyfluorescein diacetate succinimide ester; IPCs, intermediate progenitor cells; NBCs, newborn cells; RGCs, radial glial cells; SVZ, subventricular zone; VZ, ventricular zone.

We first set out to determine the proportion of IPCs and neurons derived from RGCs. We injected CFSE into the lateral ventricle, which can label the RGCs near the ventricular surface within 3 h^43^ and the newborn progenies from the RGCs. To recognize the cell identity when they enter the SVZ, determining the time window of the cohort of newborn progenies arriving at SVZ was necessary. We labelled the newborn cells generated by RGCs with CFSE at E13.5 (early embryonic stage) and E15.5 (late embryonic stage), respectively. We examined that the CFSE^+^ cells were still located in VZ at 7‐h post‐injection, and then we detected the timepoint of CFSE^+^ cells appearing in SVZ with consecutive 2‐h intervals (Supplementary Figure 1A). The results revealed that the time windows were 14 h at E13.5 and 18 h at E15.5, respectively (Figure 1A and Supplementary Figure 1B,C). Then, we performed triple‐label immunohistochemistry against CFSE, Pax6 and Tbr2. RGCs express high level of Pax6, newborn IPCs express Tbr2 and are always manifested as Pax6^+^Tbr2^+^.^15^, ^44^ Due to the fact that the newborn neurons deriving from RGCs lack appropriate marker, they were identified as CFSE^+^Pax6^−^Tbr2^−^ (Figure 1B). As depicted in our results, the ratio of IPCs was more than 90% (96.05% ± 1.14% at E13.5 and 93.62% ± 1.92% at E15.5), the proportion of direct neurogenesis was fewer than 2% (0.59% ± 0.29% at E13.5 and 1.35% ± 0.39% at E15.5) (Figure 1C). We also discovered certain bRGCs which only express Pax6 (Supplementary Figure 1D), which is consistent with previous observations.^8^, ^9^, ^10^ The percentage of bRGCs increased modestly from 3.37% ± 1.24% at E13.5 to 5.03% ± 1.55% at E15.5 (Figure 1C). Accordingly, our results suggest that more than 93% of the asymmetric RGCs division generate IPCs.

Because both IPCs and neurons are produced by the RGCs in VZ, we also analysed their proportion in this area according to the expression of transcription factors. Moreover, we utilized CD31 to identify the vascular endothelial cells because the blood vessel invaded into neocortex from E11.5 (Supplementary Figure 2A).^45^, ^46^ After confirming that the cells expressing Pax6 were all progenitor cells with Ki67 expression (Supplementary Figure 2B), neurons were identified as Hoechst^+^Pax6^−^Tbr2^−^CD31^−^ by excluding RGCs, IPCs and vascular endothelial cells (Figure 1D). In our study, we found that RGCs were the major component in VZ, and the progenies of RGCs were almost all IPCs with a tiny minority of neurons directly generated from RGCs (Figure 1E). We also uncovered that there was a slight increase in neuron production from direct neurogenesis, with the ratio from 0.72 ± 0.17% at E12.5 to 5.7 ± 0.20% at E16.5 (Figure 1F). The results suggest that IPCs constitute the vast majority of the newborn progenies from asymmetric RGCs division.

### 3.2 Majority of progenies from asymmetrical RGCs division continue to divide in SVZ

**FIGURE 2:**
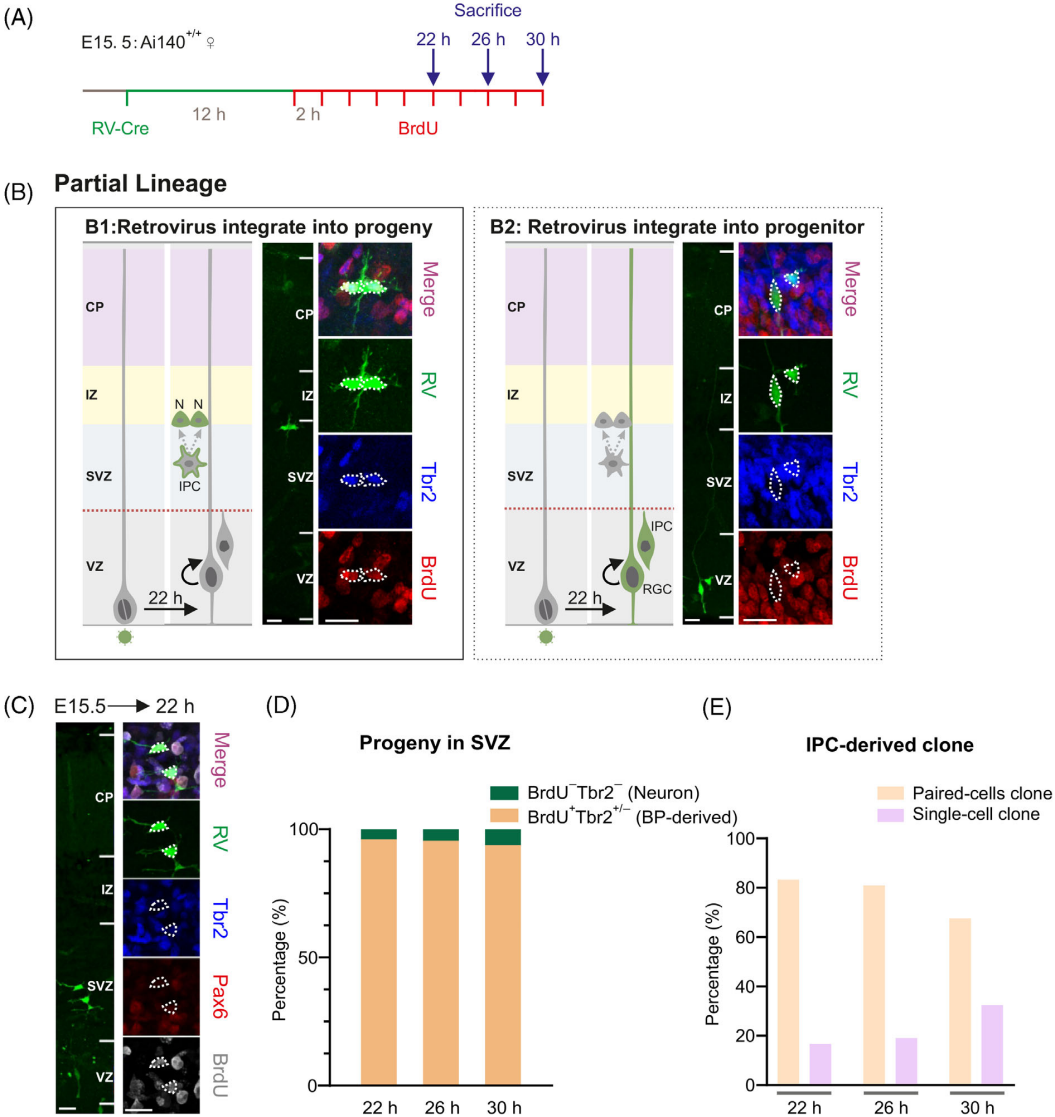
RGCs generate basal progenitor principally and partial IPC‐derived neurons undergo apoptosis immediately after birth. (A) Experimental procedure for clonal tracing and BrdU administration. (B) Schematic representation of the expected outcomes from sparse labelling in partial lineage, the two modes of partial lineage are shown in B1 and B2. If the retrovirus integrate into the progeny like B1, the progeny arrive at SVZ at 22 h after RV injection, and divide into two neurons (N) if it is an IPC. If the RV integrate into the RGC like B2, the RGC go through another cell cycle and generate a new progeny, the new progeny remain under SVZ at 22 h after retrovirus injection. Scale bar: 20 μm. (C) Representative fluorescence images of coronal section at 22 h after low‐titre Cre retrovirus injection at E15.5. High‐magnification images are represented on the right. RV co‐stained against Pax6 (red), Tbr2 (blue) and BrdU (white). Majority of paired‐cell clones disperse in a 40‐μm section and labelled by BrdU (mitotic cell). Scale bar: 20 μm. (D) Quantification of the fraction of proliferating cell (BrdU^+^) and neuron (BrdU^−^Tbr2^−^) deriving from RGCs at E15.5, with different time intervals (22, 26 and 30 h, respectively). The number of clones are 50 at 22 h, 22 at 26 h and 79 at 30 h, respectively. (E) Proportion of IPC‐derived clones containing paired cell or single cell. IPCs are from RV^+^ cells co‐labelled by BrdU, excluding bRGCs. The number of single‐cell clones increases slightly from 22 to 30 h. BP, basal progenitor; BrdU, bromodeoxyuridine; bRGCs, basal radial glial cells; IPC, intermediate progenitor cell; RGCs, radial glial cells; RV, retrovirus; SVZ, subventricular zone.

To validate that the majority of neurons are from indirect neurogenesis, we also tracked the division activity of progenies from asymmetrical RGCs division. We sparsely labelled the progenies of RGCs at E15.5 when the division cycle of RGCs is long. To detect whether the progenies continue to divide, we continuously injected BrdU every 2 h^47^ from 12‐h post‐retrovirus injection before the progenies entered the next S phase (Figure 2A). Here, we injected Cre retrovirus into the embryonic lateral ventricle of *Ai140‐EGFP* reporter mouse, the Cre‐LoxP system could effectively avoid retrovirus silencing.^48^, ^49^ The viral particles randomly enter into the renewed RGC or daughter cell (IPC or neuron) when the RGC divide asymmetrically,^33^ and we regard this renewed RGC or progeny as the first generation (G1). If the retrovirus enters the G1 progeny, the daughter cell will migrate upwards to SVZ. If the G1 progeny is an IPC, it will be labelled by BrdU and continue to divide into paired G2 neurons. If the G1 progeny is a neuron, it will never be labelled by BrdU and present as a single cell. Meanwhile, if the retrovirus enters the G1 RGC, it will continue to divide and generate a G2 RGC and a G2 progeny (IPC or neuron) in VZ (Figure 2B). Here, we focused on the situation that retrovirus labelled the G1 progeny (Figure 2B1), and collected the clones when the G1 progenies have entered into SVZ and the G2 progenies from G1 RGCs have not entered into SVZ yet. The G1 RGCs underwent a complete cell cycle (about 17.5 h at E15.5)^50^ to generate G2 progenies and the newborn G2 progenies needed another 18 h approximately to enter SVZ (Supplementary Figure 3). Thus, we collected the clones at 22, 26 and 30 h after retrovirus injection, respectively. Subsequently, we detected the BrdU in the clones (Figure 2C). We defined BrdU^+^ cells as basal progenitors (BPs, including bRGCs and IPCs) or BP‐derived offspring, and we distinguished IPCs from bRGCs by their basal process (Supplementary Figure 4A,B). We considered BrdU^−^ cells as neurons directly generated from RGCs (Supplementary Figure 4C), as they lack proliferative capacity after birth.

We analysed 50 neocortical clones from 10 embryos at 22 h after retrovirus injection. The results revealed that 96% (48/50) of clones maintained proliferative capacity (Figure 2D), and 10% (5/50) of them were bRGC‐derived clones (Supplementary Figure 4B). The bRGC division was also observed by time‐lapse microscopy (Supplementary Movie 1). Hence, more than 95% of progenies from asymmetrical RGCs division are BPs instead of neurons in clone scale.

Interestingly, we uncovered some single‐cell clones derived from IPCs (Supplementary Figure 4D), and 16.67% (8/50) of BrdU^+^ clones exhibited only one cell at 22 h (Figure 2E). We speculated that some IPC‐derived daughter cells underwent apoptosis or IPCs divided incompletely. To figure out the cause of these single‐cell clones (BrdU^+^), we analysed the percentage of single‐cell clones (BrdU^+^) at 26 and 30 h after retrovirus injection. If the ratio of the single‐cell clones decreases with the prolongation of timepoint, the phenomenon could be induced by incomplete IPCs dividing, otherwise, it could be caused by apoptosis. We discovered the ratio of the single‐cell clones increased from 19.05% at 26 h to 32.43% at 30 h instead of decreasing. The result indicates that the single‐cell clones are derived from apoptosis. Therefore, near a quarter of IPCs might undergo asymmetrical progeny apoptosis immediately after birth.

### 3.3 The neurogenetic process of individual RGC is depicted by lineage tracing

**FIGURE 3:**
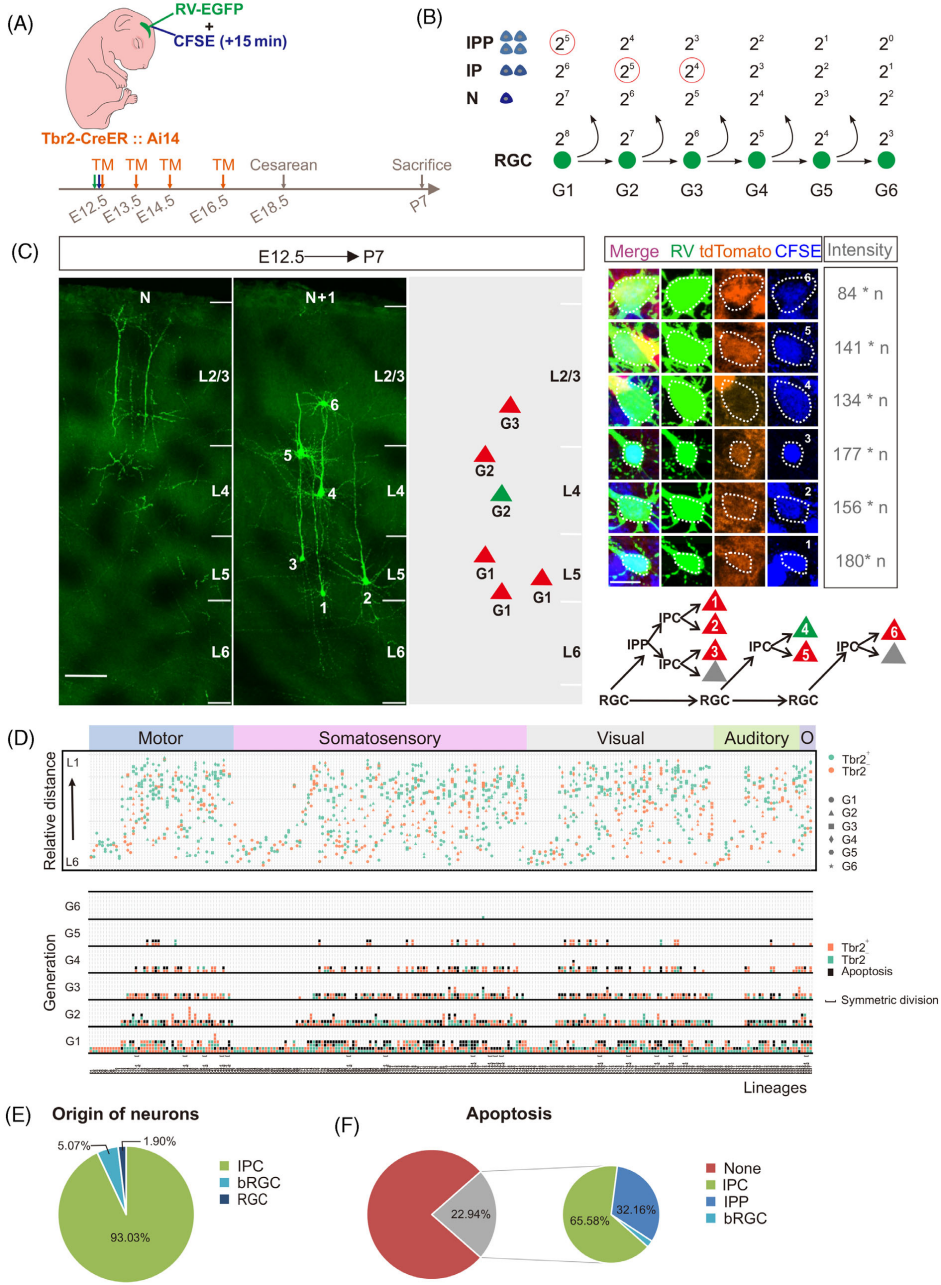
The process of RGCs differentiation presented in lineage tracing. (A) Schematic representation of the lineage tracing strategy. (B) The intensity of CFSE exponentially decreased with successive generation. (C) Confocal images and 3D reconstruction images of representative lineage at P7, high‐magnification images and the intensity of CFSE of each neuron are shown at the right, *n* = 1E + 07. The reconstituted lineage hierarchy is demonstrated at bottom right. Scale bar: 100 μm (left) and 10 μm (right). (D) Schematic dendrogram and layer distribution of 237 lineages across neocortical area (motor, somatosensory, visual, auditory and other area). Neuronal distribution is shown on top, the shape of neuron means the generation deriving from RGCs: ● means first generation (G1), ▲ means G2, ■ means G3, ♦ means G4, 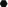 means G5, ★ means G6. The hereditary constitution of individual RGC is shown in bottom, the colour means whether labelled by *Tbr2‐CreER* mice: yes (red), no (green) or none (apoptosis, black). ∏ means symmetrical division. (E) Percentage of neuronal origin from direct neurogenesis (RGC) or indirect neurogenesis (mediated by IPC or bRGC). (F) Percentage of apoptosis and their contribution from direct neurogenesis (RGC) and indirect neurogenesis (mediated by IPC, IPP or bRGC). bRGCs, basal radial glial cells; IPC, intermediate progenitor cell; IPP, intermediate proliferative progenitor cell; RGCs, radial glial cells.

Next, to explore the neurogenetic process of individual RGC, we conducted long‐term lineage tracing using retrovirus and CFSE at E12.5 (Figure 3A). We detected the generational sequence by CFSE because the intensity of CFSE exponentially decreased with successive generation approximately.^51^, ^52^ Besides, the intensity of CFSE was associated with the division pattern in which lineage neurons were produced through IPC, IPP or direct neurogenesis (Figure 3B). In addition, the equipartition of CFSE in two daughter cells was confirmed according to the result that the intensity of CFSE was comparable between paired IPC‐derived neurons (Supplementary Figure 5A,B). To discriminate the neurons derived from direct or indirect neurogenesis, we also induced the *Tbr2‐CreER;Ai14‐tdTomato* mice via TM administration to label IPCs and IPC‐derived neurons permanently. We validated the specificity and effectiveness of *Tbr2‐CreER* mice. We observed that the tdTomato^+^ cells were located in SVZ at 14 h after TM administration, and approximately all of the tdTomato^+^ cells have entered IZ at 24 h after TM administration (Supplementary Figure 6A,B). Thus, *Tbr2‐CreER* mice never labelled RGCs, which was consistent with previous studies that RGCs definitely do not express Tbr2.^21^, ^30^ Then, we combined IdU and BrdU administration to examine the proliferative capacity of tdTomato^+^ cells. We found that more tdTomato^+^ cells were IPC‐derived neurons instead of IPCs (Supplementary Figure 6C,D). The result indicates that newborn neurons derived from IPCs express Tbr2 in short time and can be labelled by *Tbr2‐CreER* mice stochastically. So, we might label one of paired neurons derived from IPCs in lineage. Thereafter, we collected lineages at P7 when excitatory neurons have completed migration and localization,^53^ and reconstituted lineage hierarchy spanning across several sections according to the principle of closer distribution distance between the paired IPC‐derived neurons (Figure 3C).^22^, ^32^

Finally, we reconstructed 237 lineages including 58 two‐cell clones in four classic areas of neocortex from 61 brains (Figure 3D, Supplementary Diagram 1). We considered that the two‐cell clones were derived from the IPCs (Figure B1). In the remaining 179 lineages, we elaborately illustrated the neurogenetic process of individual RGC and summarized their characteristics. When we reconstructed the lineage trees, the apoptotic cells were replenished depending on the intensity of CFSE to delineate the neurogenetic process completely. Our results demonstrated that 79.33% (142/179) of RGCs contributed to the whole clones through IPCs, and 10.06% (18/179) of RGCs produced neurons directly after IPC generation. Heterogeneous RGCs generated clones with divergent proliferative potential, intermediary and layer distribution of offspring (Figure 3D and Supplementary Figure 7A,B). In addition, we found that significant difference in clone size of the lineages only existed between motor and auditory cortex (Supplementary Figure 7C), and there was no significant difference in the average generation of RGCs in different neocortical areas (Supplementary Figure 7D). The higher proliferative capacity of the RGCs in the middle neocortex corresponded to a higher proportion of RGCs that underwent symmetrical division and glial production after neurogenesis (Supplementary Figure 7E,F).

On the statistical analysis of lineage trees in neocortex, we found that the proportion of RGCs output including IPCs, bRGCs and neurons were 93.34%, 2.77% and 3.88%, respectively (Supplementary Figure 7G). Correspondingly, the neuron production from them accounted for 93.03%, 5.07% and 1.90%, respectively (Figure 3E). In conclusion, our results exhibit that the majority of neurons originate from indirect neurogenesis clearly and concretely (Supplementary Movie 2). Besides, nearly half (48.32%, 389/805) of IPCs underwent randomized apoptosis of their descendant, and apoptosis affected up to 22.94% of decrease of neuron production consequently (Figure 3F).

### 3.4 IPPs are perceived in abundance at early embryonic stage with high expression of *Cux2* in mice neocortex

**FIGURE 4:**
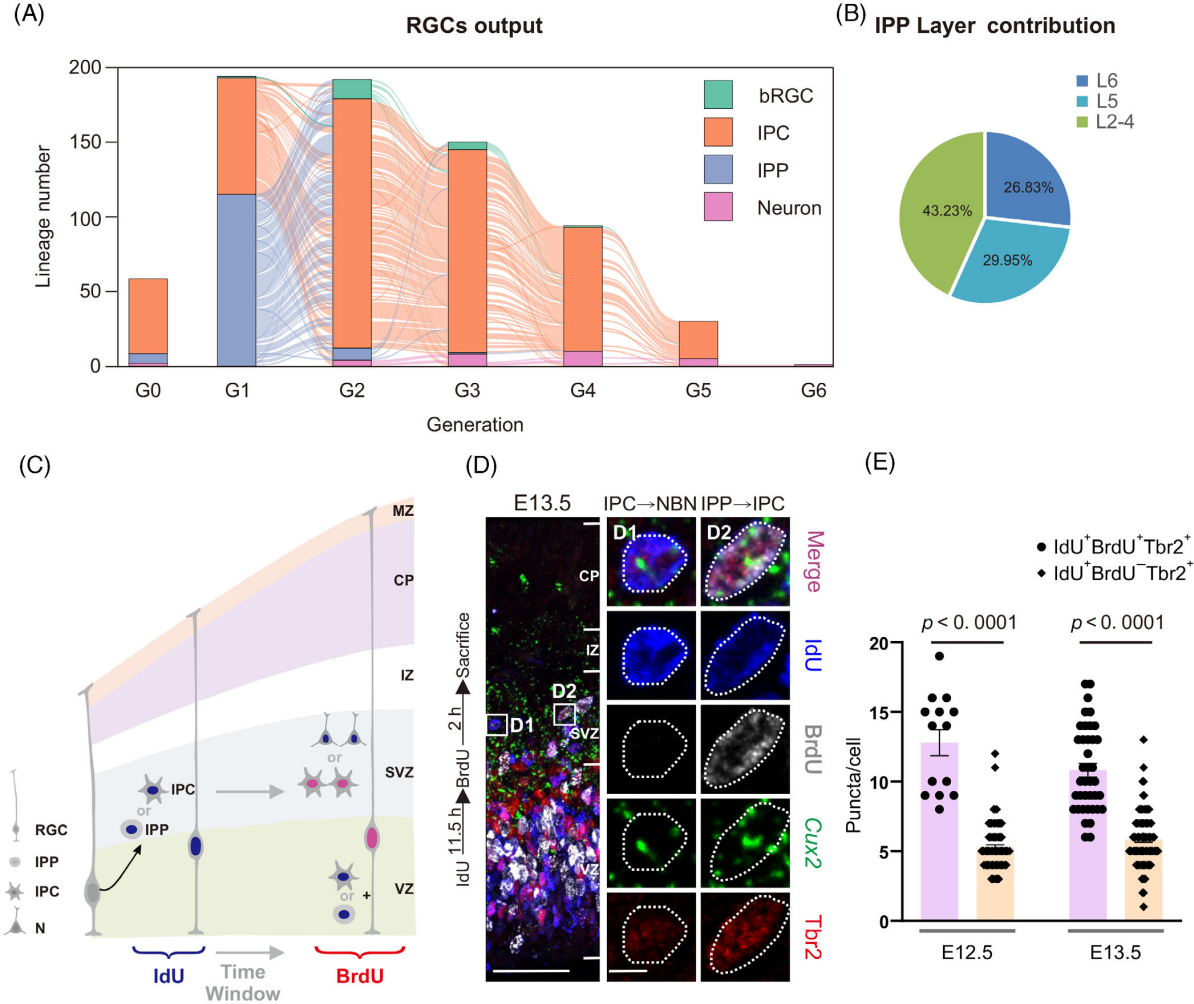
IPPs are observed at early embryonic stage and correlated with *Cux2*‐positive progenitor. (A) Alluvial plot shows the output of RGCs between different generations in a lineage. The G0 means it is the progeny that retrovirus labelled partial lineage (as shown in Figure 2B1). (B) Proportion of layer distribution about G1 IPP‐derived neurons. (C) Schematics of the experimental design for distinguishing IPP‐derived and IPC‐derived cells in SVZ using pulse‐chase IdU and BrdU. (D) RNA‐scope for *Cux2* on coronal sections of embryonic telencephalon, in the pallium. *Cux2* mRNA is mainly found in the SVZ. The sections are co‐stained with the antibodies against IdU (blue), BrdU (white) and Tbr2 (red). High‐magnification images are represented on the right. The IdU^+^BrdU^−^Tbr2^+^ cells are distinguished as IPC‐derived cells (shown in D1), and IdU^+^BrdU^+^Tbr2^+^ cells are distinguished as IPP‐derived cells (shown in D2). Scale bar: 50 μm (left) and 5 μm (right). (E) Quantification of the number of puncta in IdU^+^BrdU^+^Tbr2^+^ and IdU^+^BrdU^−^Tbr2^+^ cells in SVZ at E12.5 and E13.5. Data are mean ± SEM. Statistical significance is determined using an unpaired two‐tailed Student's *t*‐test or Mann–Whitney test. BrdU, bromodeoxyuridine; IdU, idoxuridine; IPC, intermediate progenitor cell; IPP, intermediate proliferative progenitor cell; RGCs, radial glial cells; SVZ, subventricular zone.

Interestingly, we detected considerable IPPs in lineage trees, as well as proliferative division of IPCs with time‐lapse microscopy (Supplementary Movie 1). In spite of the self‐renewability of IPCs in murine has been reported previously,^6^, ^7^, ^22^, ^29^ the proportion of IPPs and their characteristics are poorly understood. Here, we found that 19.61% (132/673) of IPCs were proliferative progenitors and the IPPs contributed to 30.66% of neocortical excitatory neurons (Supplementary Figure 7H). In addition, 66.48% (119/179) of lineages contained at least one IPP, which mainly existed in the first generation (G1) and rarely appeared in other generations (Figure 4A). We then analysed the layer distribution of neurons derived from G1 IPPs. We found that the G1 IPPs contributed to glutamatergic neurons in both deep and superficial layers, with 43.23% of them were located in superficial layers (Figure 4B). Therefore, such spatial distribution could explain the phenomenon that part of neurons derived from early IPCs are located in the upper layer eventually (Supplementary Figure 8). Overall, these results imply that IPCs possess high proliferative potential at early developmental stage, which is conducive to expand neocortex more efficiently in the limited time.

The spatial distribution of IPPs and their progenies is similar to *Cux2*‐expressing cells either embryogenetic or postnatal stage.^20^, ^54^ They are all located in SVZ at embryonic stage. At P7, 43.23% and 56.77% of G1 IPP‐derived neurons are located in superficial and deeper layers, respectively. The proportion of *Cux2*‐expressing cells in deeper and superficial layers is also roughly equivalent.^55^, ^56^ Therefore, we speculated that IPPs were probably the *Cux2*‐expressing progenitors in SVZ.

To further consolidate our hypothesis, we compared the expression of *Cux2* between IPP‐derived cells and non‐proliferative IPC‐derived cells. We conducted an experiment with hybridizing *Cux2* in situ in two groups of cells distinguished by IdU/BrdU injection (Figure 4C). Firstly, we administrated IdU intraperitoneally at early embryonic stage (E12.5 and E13.5). Then, we injected BrdU after an approximate cell cycle (10 h at E12.5 and 11.5 h at E13.5)^50^ and sacrificed 2 h later. The uridine analogues specifically labelled proliferative cells at S phase, thus, IdU labelled the progenitors including RGCs in VZ, IPPs and IPCs in SVZ. Admittedly, few bRGCs were probably labelled by IdU. We observed IdU^+^BrdU^+^ and IdU^+^BrdU^−^ cells in SVZ which were derived from IPPs and IPCs, respectively. Meanwhile, IdU^+^BrdU^+^ and IdU^+^BrdU^−^ cells in VZ were RGCs and newborn IPCs, respectively (Supplementary Figure 9A,B). To exclude that the IdU^+^BrdU^+^ cells in SVZ were the IdU^+^BrdU^−^ newborn IPCs which migrated from VZ and re‐entered next S phase, we tested the time window of IdU^+^ RGCs from S phase to M phase at first.^57^, ^58^ We injected CFSE into E13.5 ventricle to label the RGCs in M phase, and administrated IdU to mark the RGCs in S phase 4 or 7 h before. Then we analysed whether CSFE^+^ cells were co‐labelled with IdU at 14 h after CFSE injection. We found that partial CFSE^+^ cells co‐labelled with IdU when the time window was 4 h, while they were co‐labelled entirely when the time window was 7 h (Supplementary Figure 9C–E). Thus, the time window of IdU^+^ RGCs from S phase to M phase is approximately 7 h at E13.5. Besides, to detect whether the IdU^+^BrdU^−^ newborn IPCs from RGCs entered S phase before they leave VZ, we examined IdU^+^Tbr2^+^ cells at the VZ/SVZ boundary while none in VZ at 2 h after IdU injection (Supplementary Figure 9F,G). Thus, the results validate that the newborn IPCs were never labelled by IdU/BrdU during the stage migrated to SVZ. In conclusion, the newborn IdU^+^BrdU^−^ IPCs remain in VZ during our observation, and the IdU^+^BrdU^+^ cells exist in SVZ at the beginning. Ultimately, we can distinguish the IPP‐derived and IPC‐derived cells, although some IPCs stem from IPPs potentially.

We examined the abundance of *Cux2* between the IdU^+^BrdU^+^ and IdU^+^BrdU^−^ cells in SVZ using fluorescence in situ hybridization, along with co‐immunostaining against Tbr2. Consequently, our experiments demonstrated that *Cux2* was primarily expressed in SVZ (Figure 4D), and the number of *Cux2* puncta in per cell between two groups of cells was discrepant (Figure 4E). In detail, the average number of puncta in per cell was double in IdU^+^BrdU^+^ cells compared to IdU^+^BrdU^−^ cells within E12.5 and E13.5. These results suggest that IPPs express high level of *Cux2* characteristically compared to non‐proliferative IPCs. The results also imply that the IPCs expressing *Cux2* may possess higher proliferative potential, and the hypothesis needs to be further experimentally validated.

### 3.5 The neurogenetic process of individual RGC and cell identity are unaffected in *Reeler* mice

To detect whether the differentiation process of RGCs is controlled by endogenous or exogenous signals, we introduced *Reeler* mouse with abnormal Reelin expression. Reelin, an extracellular matrix protein secreted by Cajal Retzius (CR) cells located in the marginal zone (MZ), controls the downstream signalling pathway to affect the radial glial scaffold and normal neocortex formation.^59^, ^60^, ^61^ Reelin deletion leads to disorganized neocortical structures and modifies the proliferation dynamics of RGCs (Supplementary Figure 10).^62^, ^63^ Thus, we conducted lineage tracing in *Reeler* mice to investigate whether their RGCs differentiation process is conservative.

**FIGURE 5:**
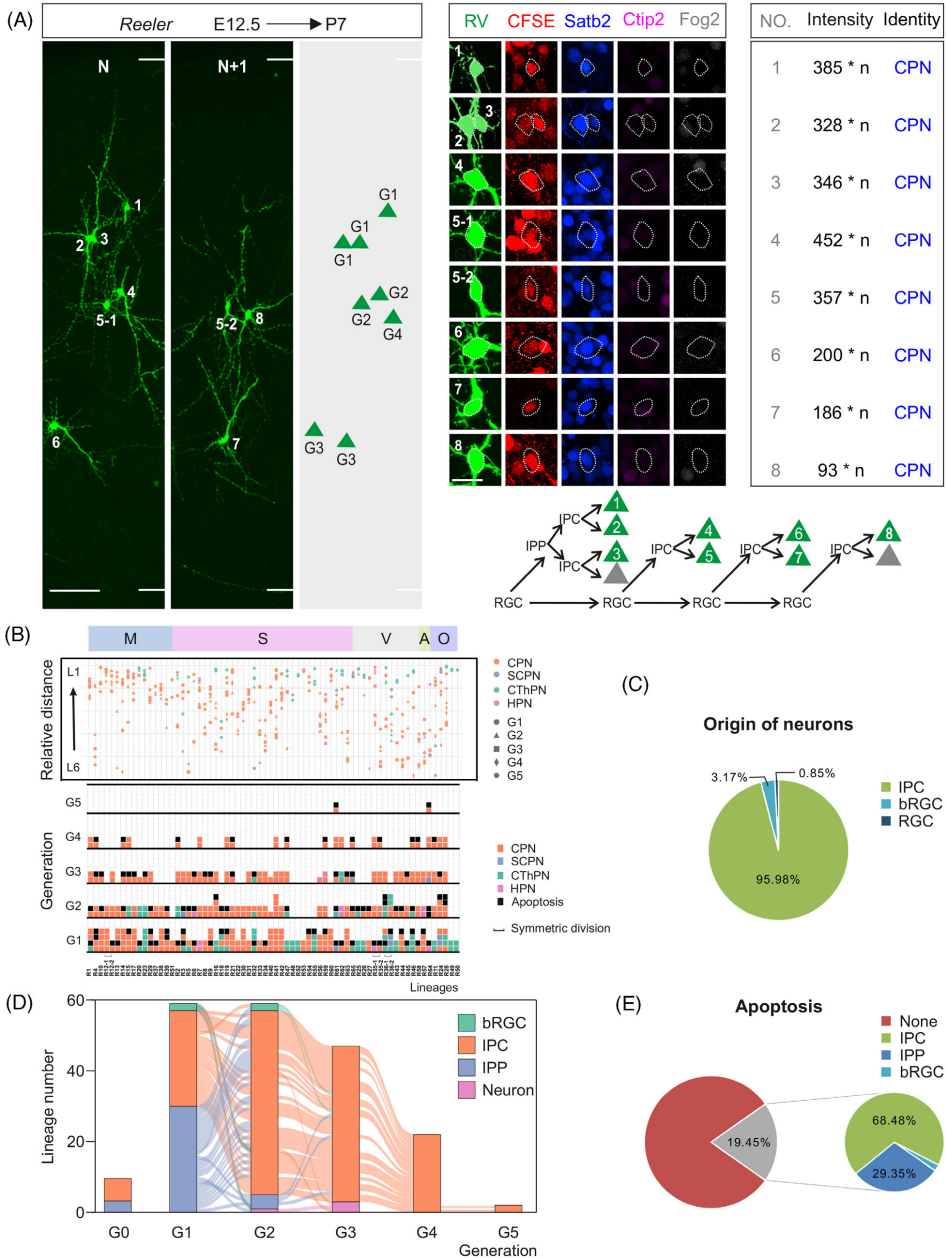
The process of RGCs differentiation presented in *Reeler* mice. (A) Confocal images and 3D reconstruction images of representative lineage in *Reeler* mice, high‐magnification images of each neuron are shown at the right, the neuron of NO.5 was cut and distributed to adjacent slices. The intensity of CFSE of each neuron and the identity of subtype are shown at the right, *n* = 1E + 06. The reconstituted lineage hierarchy is demonstrated at bottom right. Scale bar: 100 μm (left) and 20 μm (right). (B) Schematic dendrogram and layer distribution of 65 lineages in motor cortex (M), somatosensory cortex (S), visual cortex (V), auditory cortex (A) and other cortex (O). Neuronal distribution is shown on top, the shape of neuron means the generation deriving from RGCs: ● means first generation (G1), ▲ means G2, ■ means G3, ♦ means G4, 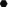 means G5. The hereditary constitution of individual RGC is shown in bottom, the colour means the type of neuron: CPN (yellow), SCPN (wathet), CThPN (green), HPN (pink) or none (apoptosis, black). ∏ means symmetrical division. (C) Percentage of neuronal origin from direct neurogenesis (RGC) or indirect neurogenesis (mediated by IPC or bRGC). (D) Alluvial plot shows the output of RGCs between different generations in *Reeler* lineage. (E) Percentage of apoptosis and their source from direct neurogenesis (RGC) and indirect neurogenesis (mediated by IPC, IPP or bRGC). bRGC, basal radial glial cell; CPN, callosal projection neuron; CThPN, corticothalamic projection neuron; HPN, heterogeneous projection neurons; IPC, intermediate progenitor cell; IPP, intermediate proliferative progenitor cell; RGCs, radial glial cells; SCPN, subcerebral projection neuron.

We traced the differentiation process of individual RGC using retrovirus and CFSE as mentioned above in *Reeler* mice. Meanwhile, we employed classic markers, Satb2, Ctip2 and Fog2 to identify the subtype of neocortical PNs in *Reeler* lineage (Figure 5A).^64^ There are three main subtypes of neocortical PNs, including callosal PN (CPN, Satb2^+^), subcerebral PN (SCPN, Ctip2^+^) and corticothalamic PN (CThPN, Fog2^+^).^65^ Besides, a group of neurons that co‐express Ctip2 and Satb2 may be CPNs or SCPNs,^66^ we collectively categorized them as heterogeneous PNs (HPNs). We reconstructed 65 lineages including 9 two‐cell clones in *Reeler* neocortex from 10 embryos (Figure 5B and Supplementary Diagram 2). Similar to the wild‐type mice, 89.29% (50/56) of RGCs contributed to the whole clones through IPCs in homozygous mutant mice. The average clone size (7.6 vs. 8.9) and generation (3.2 vs. 3.5) of lineages in *Reeler* mice and in wild‐type demonstrated that the proliferative potential of RGCs was unaffected in *Reeler* mice (Supplementary Figure 11A,B).

We found that the proportion of RGCs output including IPCs, bRGCs and neurons were 95.96%, 2.02% and 2.02%, respectively (Supplementary Figure 11C). Correspondingly, the neuron production from them accounted for 95.98%, 3.17% and 0.85%, respectively (Figure 5C). Besides, 19.47% (37/190) of IPCs underwent a round of proliferation before neurogenic division and the IPPs contributed to 31.29% of neuron production eventually (Supplementary Figure 11D). 51.78% (29/56) of lineages contained at least an IPP, the IPPs mainly existed in the G1 and seldom appeared in other generations (Figure 5D). In conclusion, our results suggest that the process of RGCs differentiation in *Reeler* mice is similar to the neurogenetic process under normal circumstances. Besides, the percentage (19.45%) of apoptotic cells in *Reeler* mice was almost comparable to the 22.94% in wild‐type mice (Figure 5E). Each subtype of PNs was observed in lineage neurons, and the majority of neurons were CPNs (Figure 5B). We delineated the differentiation process and neuron identity of heterogeneous lineages without the born time of neurons. Because the neuron subtype is associated with born time,^67^ we cannot ascertain whether the differentiated fate of neurons is changed in the *Reeler* mice.

**FIGURE 6:**
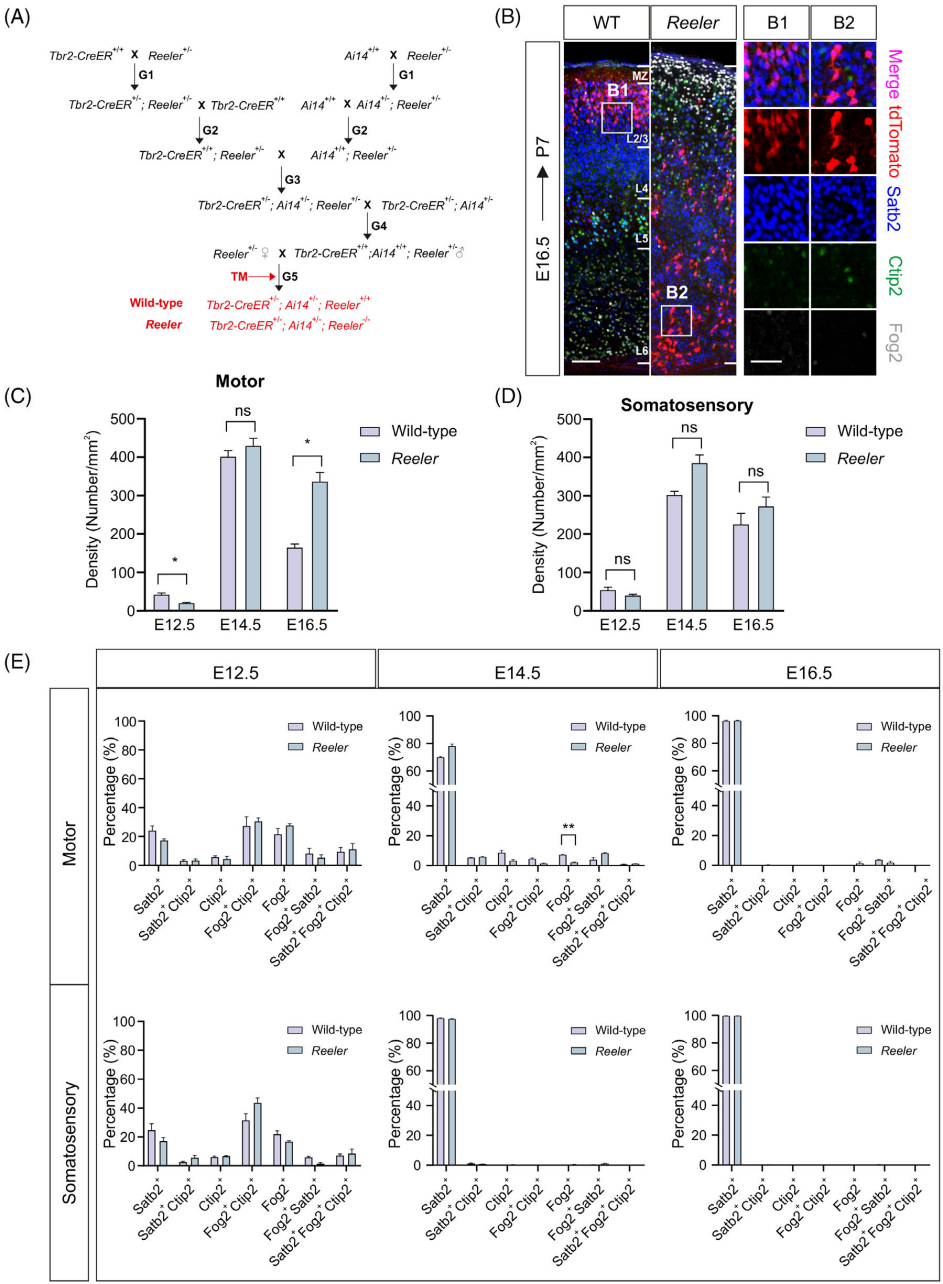
The developmental fate of neurons is conserved in *Reeler* mice. (A) Illustration of mouse breeding strategy to label the IPC‐derived neurons fluorescently in littermates of wild‐type and Reeler mice. (B) The layer distribution and identity of neurons labelled by *Tbr2‐CreER;Ai14* line in normal and Reeler mice at E16.5. High‐magnification images are shown at the right. Scale bar: 100 μm (left) and 50 μm (right). (C, D) The density of IPC‐derived neurons in motor cortex (C) and somatosensory cortex (D) at E12.5, E14.5 and E16.5, respectively. (E) The percentage of neuron types with different molecular expression in normal and Reeler mice at E12.5, E14.5 and E16.5, respectively. Data are mean ± SEM. Statistical significance is determined using two‐way ANOVA. *p < 0.05, **p < 0.01, and ns means not significant. ANOVA, analysis of variance; IPC, intermediate progenitor cell.

To detect whether the fate of PNs is conserved, we characterized the identity of IPC‐derived neurons in normal and abnormal neocortex. We established the inducible *Tbr2‐CreER* ^*+/−*^ *;Ai14* ^*+/−*^ *;Reeler* ^*+/+*^ (wild‐type) and *Tbr2‐CreER* ^*+/−*^ *;Ai14* ^*+/−*^ *;Reeler* ^*−/−*^ (*Reeler*) mouse lines (Figure 6A) allowing us to fluorescently label IPC‐derived neurons at specific embryonic stages (Figure 6B). We intraperitoneally administered TM to pregnant mice at E12.5, E14.5 and E16.5, respectively. Then, we compared the density and marker expression of the IPC‐derived neurons in motor and somatosensory cortex of *Reeler* and wild‐type littermates at P7. We found that the density of IPC‐derived neurons was lower in *Reeler* motor cortex at E12.5 while higher in *Reeler* motor cortex at E16.5, and there was no significant difference in the motor cortex at E14.5 and somatosensory cortex (Figure 6C,D). In addition, we found that the differences of marker expression between *Reeler* and wild‐type mice were not significant (Figure 6E). The results indicate that the developmental fate of PNs is unaffected at different embryonic timepoints and neocortical areas in *Reeler* mice. Thus, the differentiation pattern, proliferative potential and neuronal fate of RGCs are conserved in *Reeler* mice.

## 4 DISCUSSION

Recently, some studies suggest that IPCs generate possibly all of the PNs in the neocortex,^16^, ^68^ but lack sufficient experimental evidence. In this study, we systematically analysed the percentage of direct neurogenesis and indirect neurogenesis in meso and clone scale within the neocortex. Besides, we analysed the differentiation process of RGCs by lineage tree reconstruction. The results suggest that 79.33% of RGCs contribute to the whole clones through IPCs, and 93.03% of neurons (including apoptotic cells) derive from IPCs. Besides, half of the lineages generate an IPP at first generation with high‐level *Cux2* expression. Finally, we validated the differentiation process and characteristics of neocortical lineages are conserved in *Reeler* mice and they are probably determined by endogenous signals of RGCs.

Our results suggest that most of the glutamatergic neurons are born from IPCs and few of them are from bRGCs and RGCs. With respect to SNP (also called aIPC), a type of progenitor cell resided in VZ and independent with RGCs and IPCs.^21^, ^26^, ^69^ Actually, we barely observed schismatic SNP adjacent to ventricular surface of embryonic cerebral cortex from E12.5 to E16.5 (Figure 1D), they are probably IPCs newly generated by RGCs.^16^, ^44^ The neurogenetic contribution of IPCs was always underestimated previously by analysing clone size only. Perhaps, the phenomenon is attributed to omissive apoptosis of neurons, because we validate that a quarter of neurons undergo stochastic apoptosis (Figure 3F). This is one of the reasons why the proportion of indirect neurogenesis in our lineage is higher than the result reported by Huilgol et al. recently.^70^ They utilized the genetic intersection–subtraction strategy to detect the proportion of neurons from direct and indirect neurogenesis at P30, and collected the neurons without apoptotic neurons. Indeed, IPCs express several “dependence receptors” that induce apoptosis in the absence of ligand, such as Ntrk3, Unc5d and Epha4.^16^ In the end, the cells died procedurally in developing neocortex are phagocytosed by microglia.^71^, ^72^, ^73^ Actually, previous studies have indicated that apoptosis occur in embryonic SVZ utilizing MADM system^22^ and ‘in situ end‐labelling’^74^ instead of caspase‐3 staining. The apoptotic cells are often eliminated in short moment lasting from a few minutes to a maximum of 3 h to avoid inflammatory response.^74^, ^75^, ^76^ Compared to embryonic stage, apoptosis is detected infrequently after birth.^32^ In summary, rapid expansion of cerebral neocortex relies immensely on IPC‐mediated indirect neurogenesis,^77^ and apoptosis may regulate the totality of neocortical neurons and the proportion of subtypes.

IPCs not only amplify the number of neocortical PNs, but also molecularly conduct the axogenesis and excitability of PNs as they specifically express associate genes, such as *Pcdh7*, *Nrn1* and *Fgf11*.^16^ Single‐cell profiling described that gene clusters involved in specifying neuronal fate have been expressed in the IPCs earlier.^37^, ^39^, ^44^ In addition, IPCs guide the INs tangential migration and stimulate microglia proliferation by secreting chemokine *Cxcl12*.^71^, ^78^, ^79^ Tbr2, a characteristic marker of IPCs, directly regulates the IPCs differentiation instead of IPCs generation during early neurogenesis.^21^, ^80^ *Tbr2* mutation in humans causes microcephaly with polymicrogyria, which indicates that IPCs differentiation is necessary to drive gyrification.^81^

Our experiments implicate that the early IPCs possess higher proliferative potential while late IPCs do not (Figure 4A). Besides, the IPPs express high‐level *Cux2* compared to non‐proliferative IPCs (Figure 4E). *Cux2* is expressed in SVZ progenitors and their descendants in cortical plate, especially in CPNs in both deep and superficial layers.^20^, ^54^ Thus, *Cux2* may potentially act as an intrinsic regulatory factor to facilitate IPCs proliferation and CPN production. Currently, the regulatory mechanism underlying the high proliferative potential in early IPCs remains poorly understood. Delta–Notch signalling plays a crucial role in proliferation, because IPCs are the major source of Dll1.^44^, ^82^, ^83^ Further research is needed to determine their functions in regulating the proliferation of IPCs. On the whole, our results indicate that there are quite a few IPPs with higher *Cux2* expression in the early rodent neocortex. This finding provides a new insight into IPC proliferation.

Our study provides the first analysis on the differentiation process with temporal, spatial and hierarchical information of individual RGC. Ultimately, the results display the pattern of neurogenesis in lineage scale and validate the conservation of RGC differentiation in *Reeler* mouse. In addition, we found that 7.26% (13/179) and 26.79% (15/56) of lineages generated astrocytes after glutamatergic neurogenesis in wild type and *Reeler* mouse, respectively. Previous study has reported that the elimination of CR cells accelerates the transformation of RGCs into astrocytes.^84^ To detect whether the neurogenesis is terminated in advance in *Reeler* mice, we analysed the neuron production in the lineages contained gliocyte. The results showed that there was no significant decrease in average clone size (8 vs. 7.6) and generation (3.25 vs. 3.2). Moreover, we traced bRGCs in 20 lineages, and they tended to divide two or three times (Supplementary Movie 1), which indicates that bRGCs are not only present in gyrencephalic brains.

Indeed, there are some limitations in our study. First of all, retrovirus might be silenced in lineage tracing. However, the phenomenon was rare in our study because the overall ratio of neuronal decrease was equivalent to the percentage of apoptotic cells in Cre‐LoxP system (Figure 2). Second, retrovirus labelled some INs that invaded the cerebral cortex following tangential migratory routes. The INs were obviated according to their morphological characteristics and enormous CFSE intensity. Third, we could not recognize all IPC‐derived neurons in virtue of *Tbr2‐CreER* mice due to the dose‐dependent effect of TM. Therefore, we reconstructed the lineage trees incorporated with the information of CFSE intensity. Finally, we could not track the progeny if they migrate out of the neocortex. The problems will be solved with technological innovations in the future. Actually, accurate neurogenesis in neocortex is the basis of neural circuit assembly and functional column formation. This study provides a further understanding of neurogenesis in rodents, but it remains to be explored that the intrinsic molecular mechanisms about progenitor characteristics and their differentiation.

## AUTHOR CONTRIBUTIONS

Huan‐Huan Deng and Yinghui Fu designed the experiments. Huan‐Huan Deng performed the experiments, collected the data and prepared the figures. Shi‐Yuan Tong performed the bioinformatic analysis. Dan Shen and Shu‐Qing Zhang contributed to the methods/materials. Yinghui Fu and Huan‐Huan Deng drafted the manuscript.

## CONFLICT OF INTEREST STATEMENT

The authors declare no conflicts of interest.

## Supporting information


**Data S1.** Supporting Information.

## Data Availability

The data that support the findings of this study are available in the supplementary material of this article.
