# Supplementary material for: The characteristics of excitatory lineage differentiation and the developmental conservation in Reeler neocortex
Source: Cell Prolif. 2023 Dec 12;57(5):e13587. doi: 10.1111/cpr.13587 (PMC11056708; doi:10.1111/cpr.13587)
Supplement: Supplementary file 1 — Data S1. Supporting Information. [file CPR-57-e13587-s001.zip › Supplementary diagram 1.pdf]

Motor

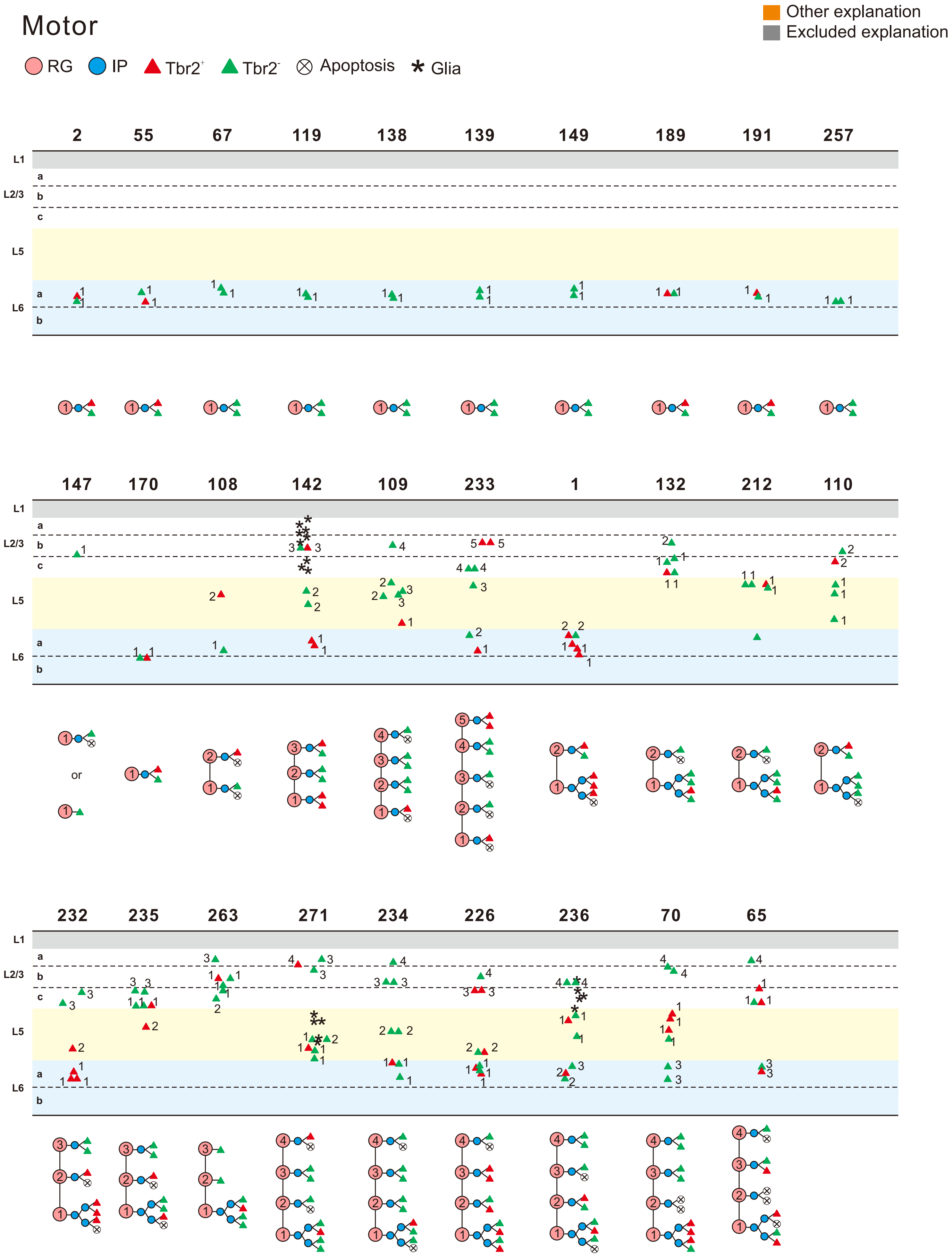
Other explanation
Excluded explanation
RG
IP
Tbr2+
Tbr2-
Apoptosis
Glia
2 55 67 119 138 139 149 189 191 257
147 170 108 142 109 233 1 132 212 110
232 235 263 271 234 226 236 70 65
L1
L2/3
L5
L6
a
b
c
or

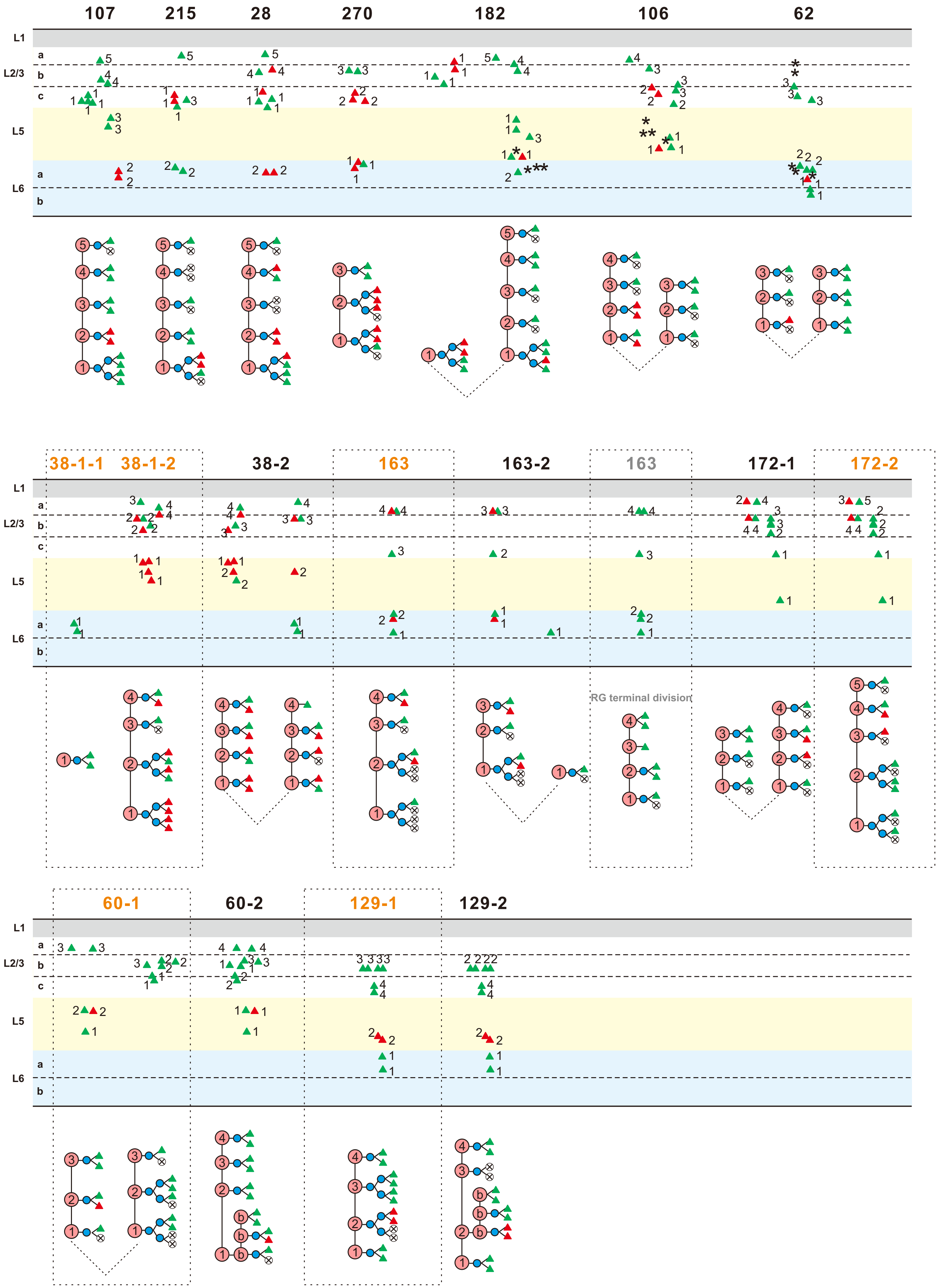
107
215
28
270
182
106
62
L1
L2/3
L5
L6
a
b
c
38-1-1
38-1-2
38-2
163
163-2
163
172-1
172-2
RG terminal division
60-1
60-2
129-1
129-2

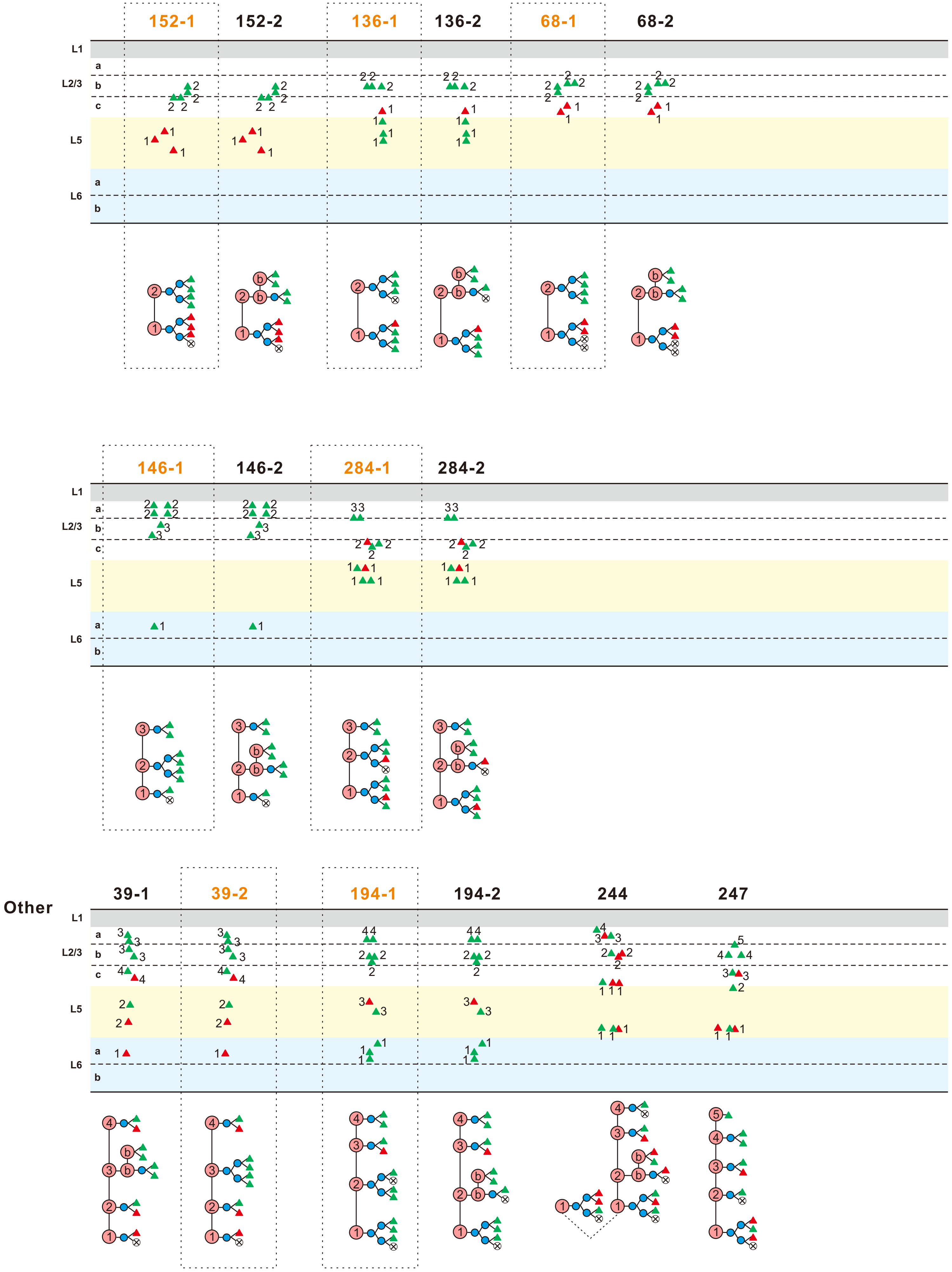
152-1
152-2
136-1
136-2
68-1
68-2
L1
L2/3
L5
L6
146-1
146-2
284-1
284-2
Other
39-1
39-2
194-1
194-2
244
247

Somatosensory

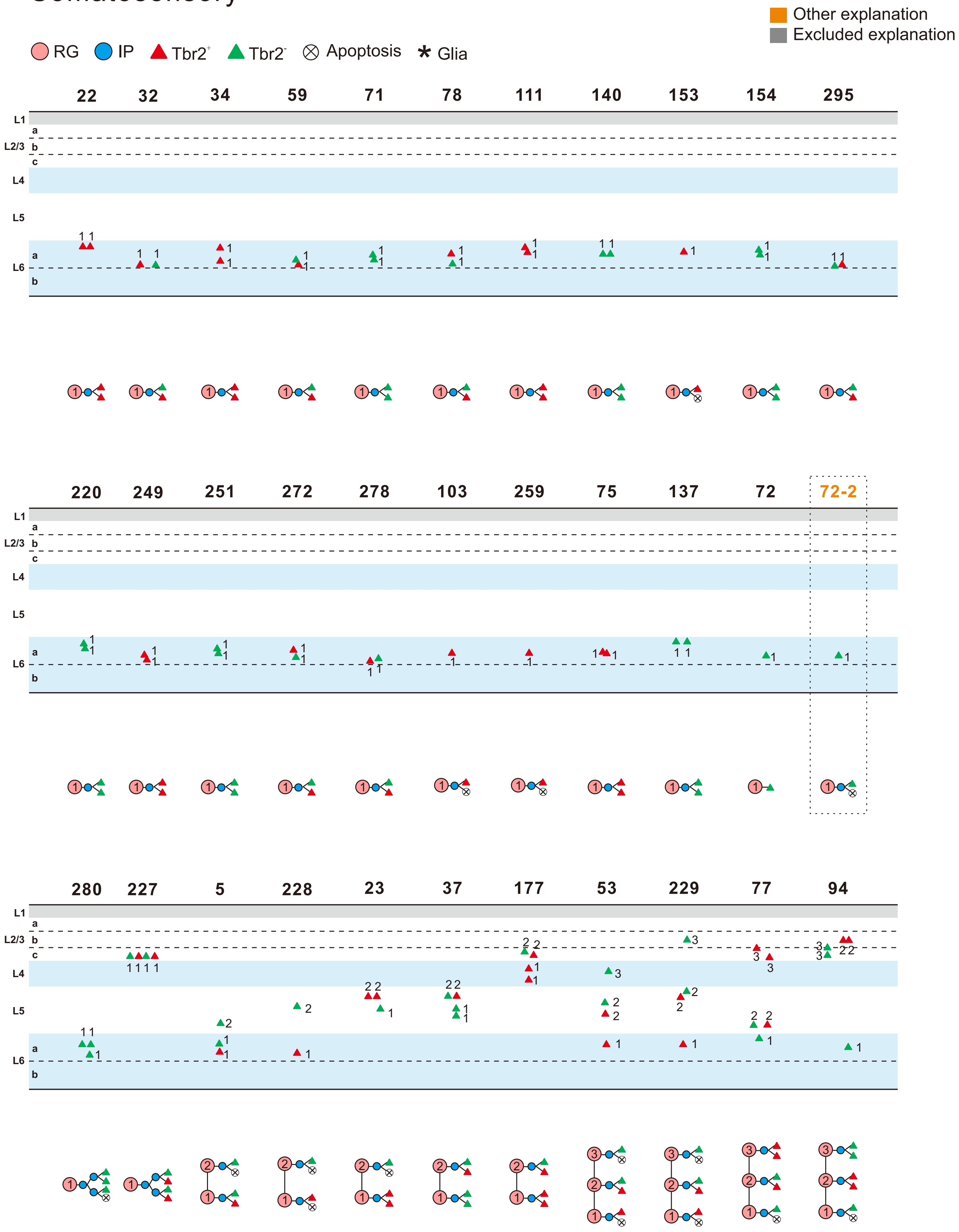

Somatosensory

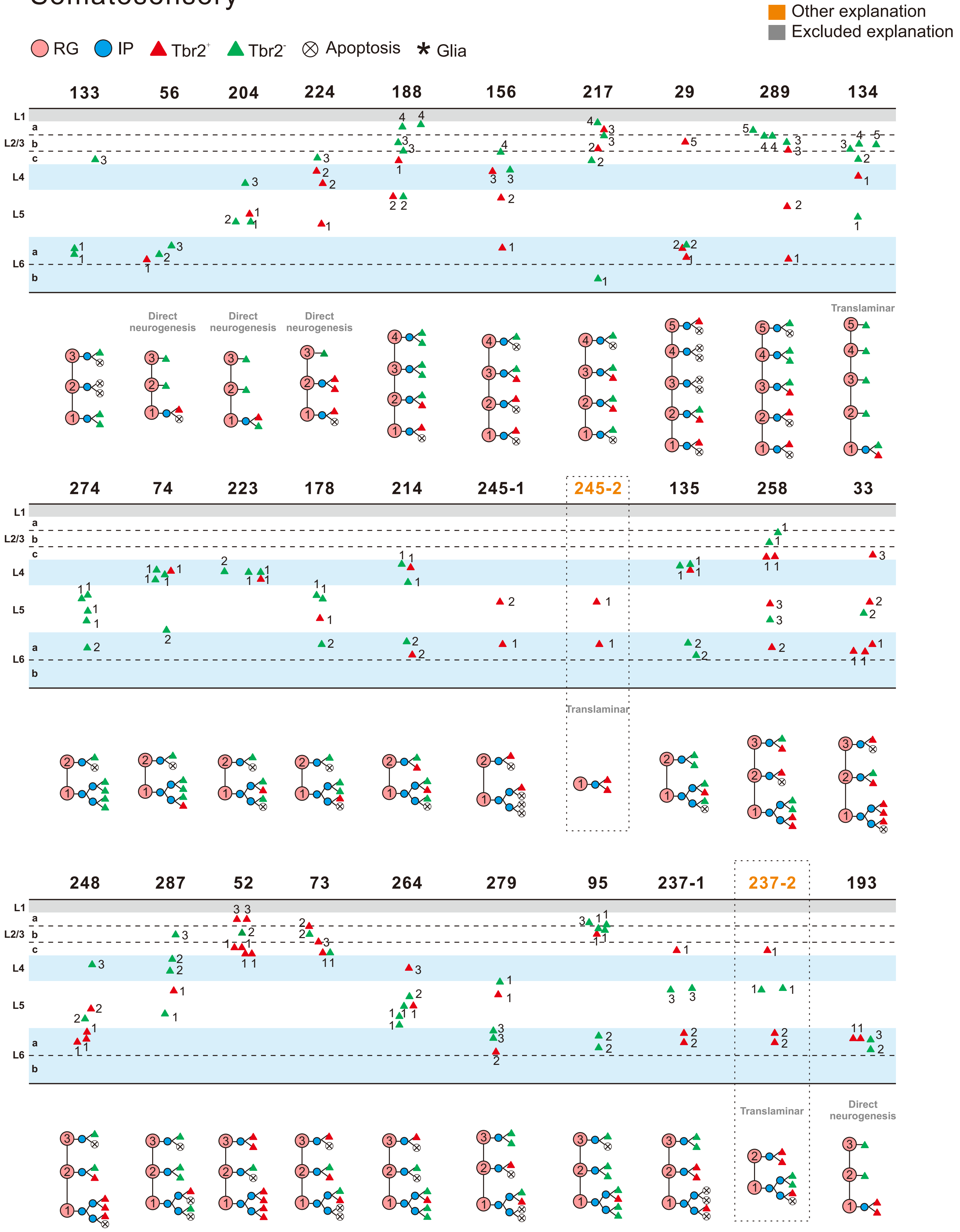

Somatosensory

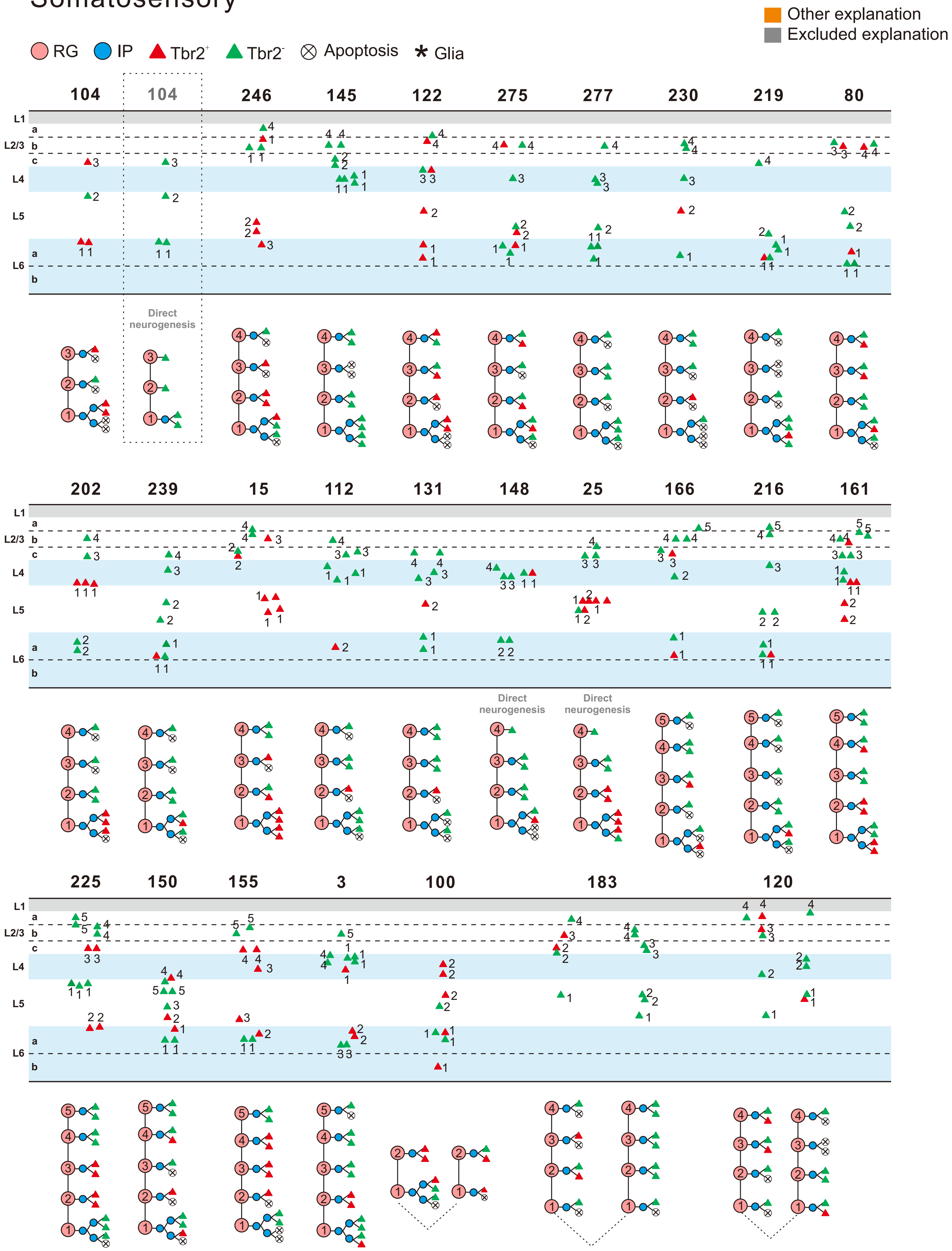

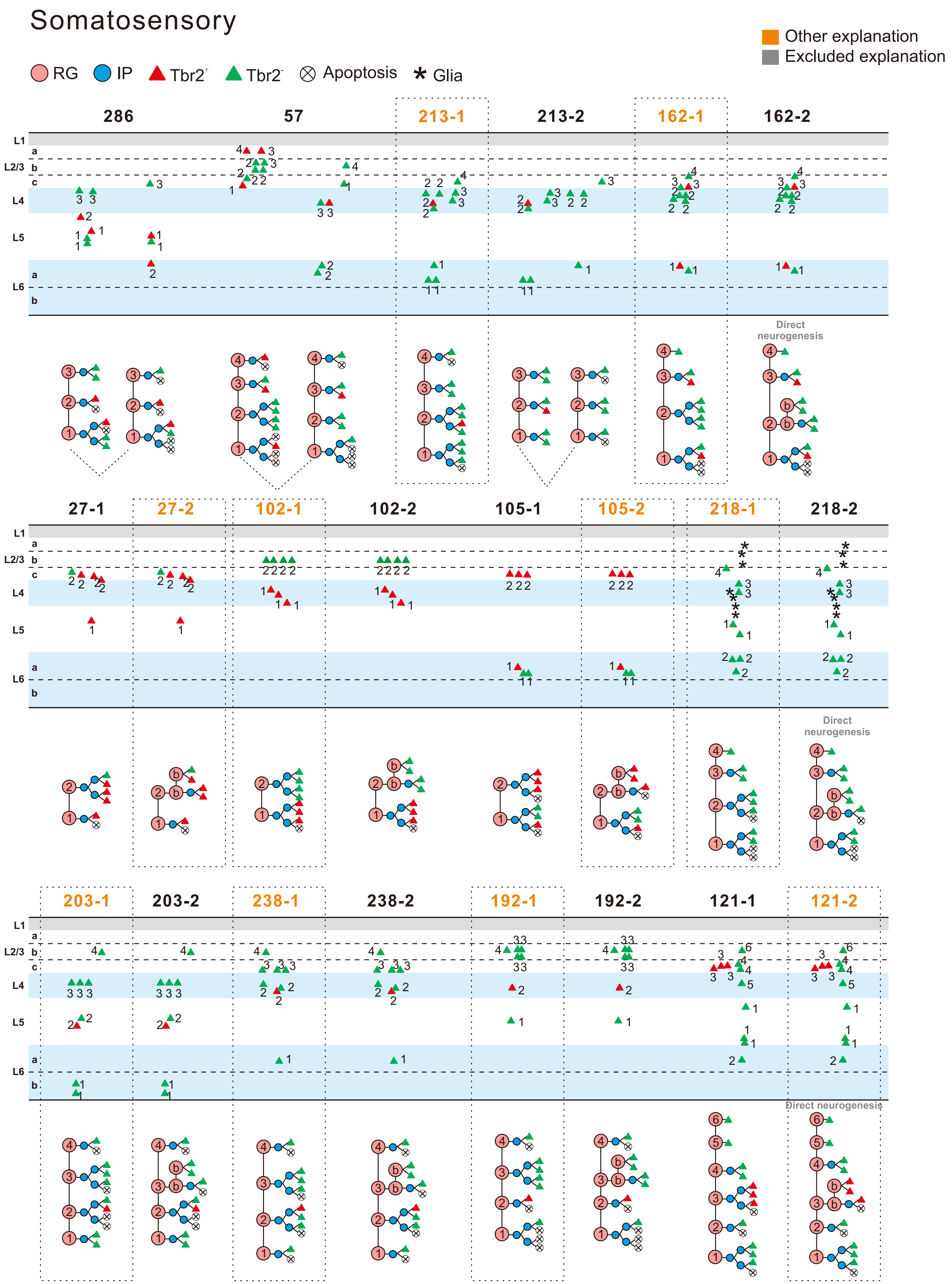
Somatosensory
RG
IP
Tbr2+
Tbr2-
Apoptosis
Glia
Other explanation
Excluded explanation
286
57
213-1
213-2
162-1
162-2
27-1
27-2
102-1
102-2
105-1
105-2
218-1
218-2
203-1
203-2
238-1
238-2
192-1
192-2
121-1
121-2
L1
L2/3
L4
L5
L6
Direct neurogenesis

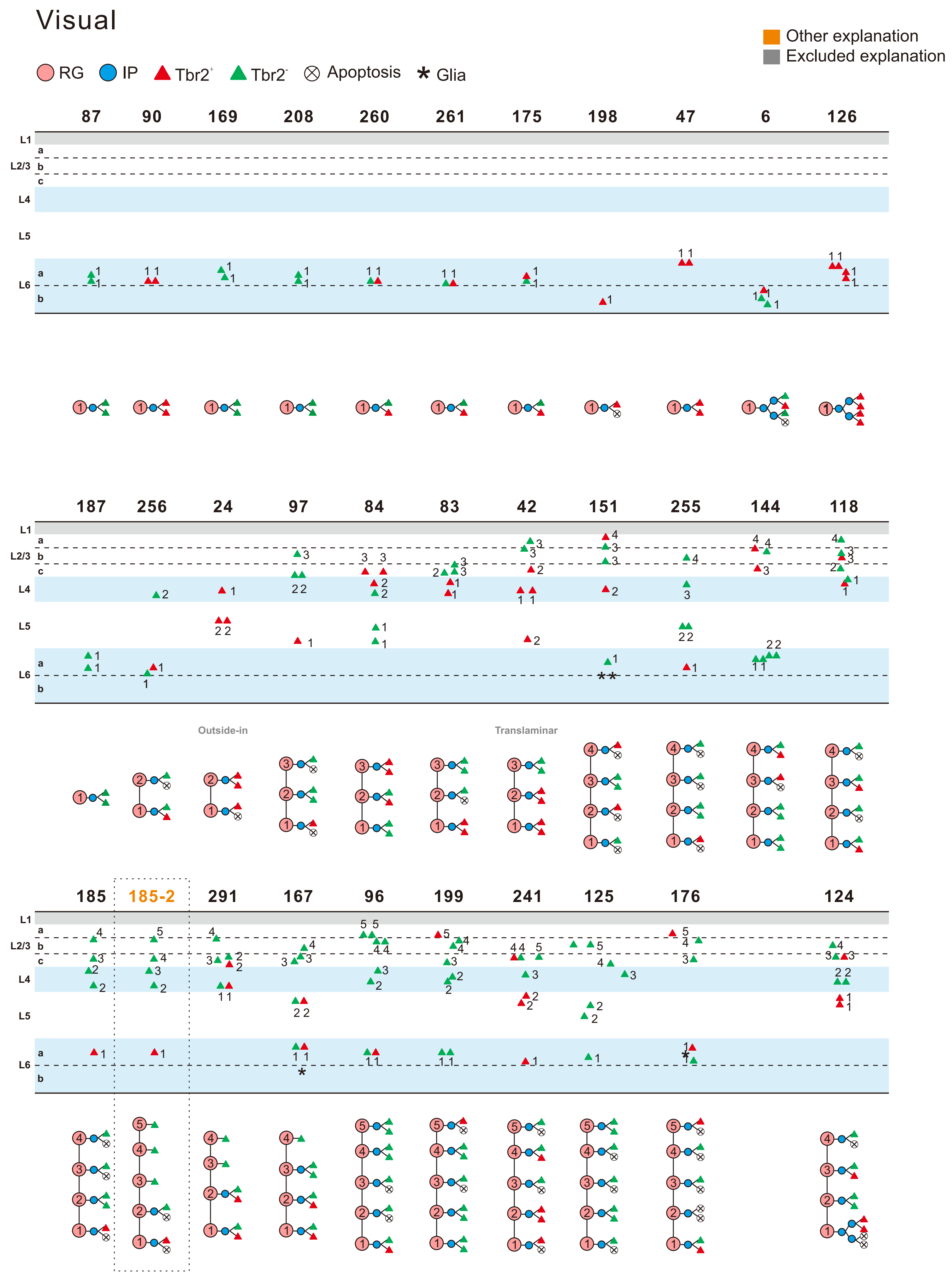
Visual
Other explanation
Excluded explanation
RG
IP
Tbr2+
Tbr2-
Apoptosis
Glia
87 90 169 208 260 261 175 198 47 6 126
L1
L2/3
L4
L5
L6
187 256 24 97 84 83 42 151 255 144 118
Outside-in
Translaminar
185 185-2 291 167 96 199 241 125 176 124

Visual

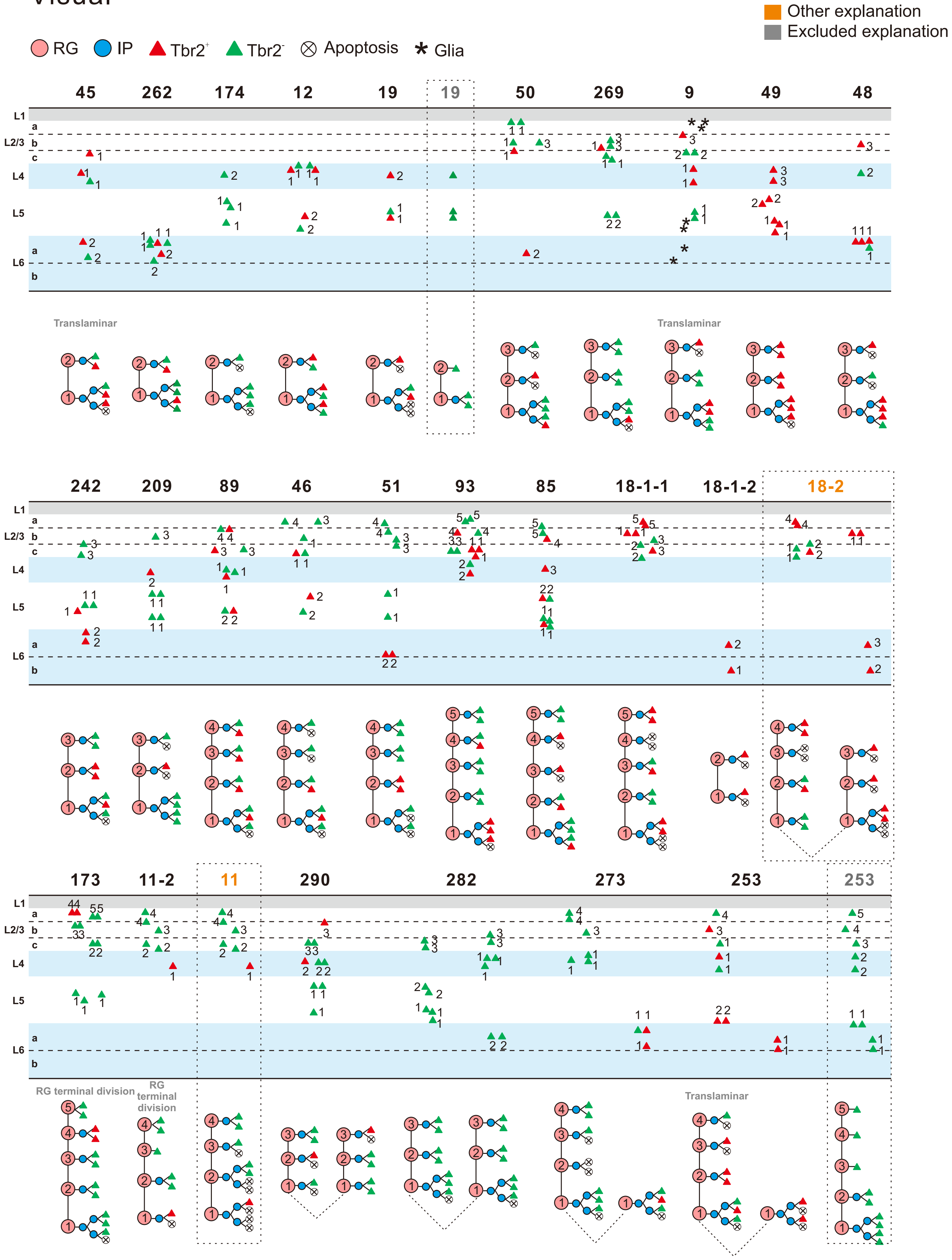

Other explanation
Excluded explanation
RG
IP
Tbr2+
Tbr2-
Apoptosis
Glia
45 262 174 12 19 19 50 269 9 49 48
242 209 89 46 51 93 85 18-1-1 18-1-2 18-2
173 11-2 11 290 282 273 253 253
L1
L2/3
L4
L5
L6
Translaminar
RG terminal division
RG terminal division

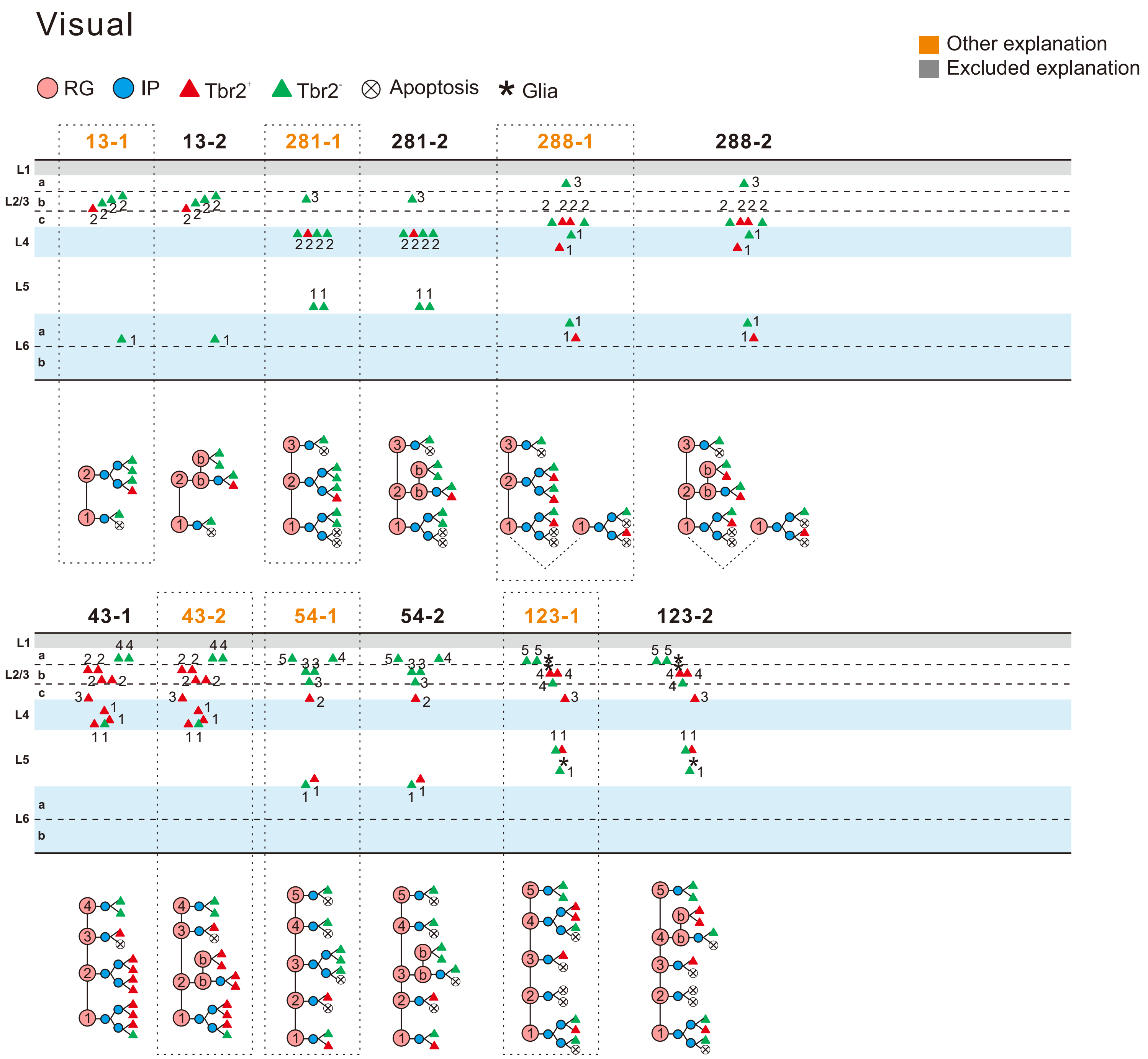

Visual
Other explanation
Excluded explanation
RG
IP
Tbr2+
Tbr2-
Apoptosis
Glia
13-1
13-2
281-1
281-2
288-1
288-2
43-1
43-2
54-1
54-2
123-1
123-2
L1
L2/3
L4
L5
L6

Auditory

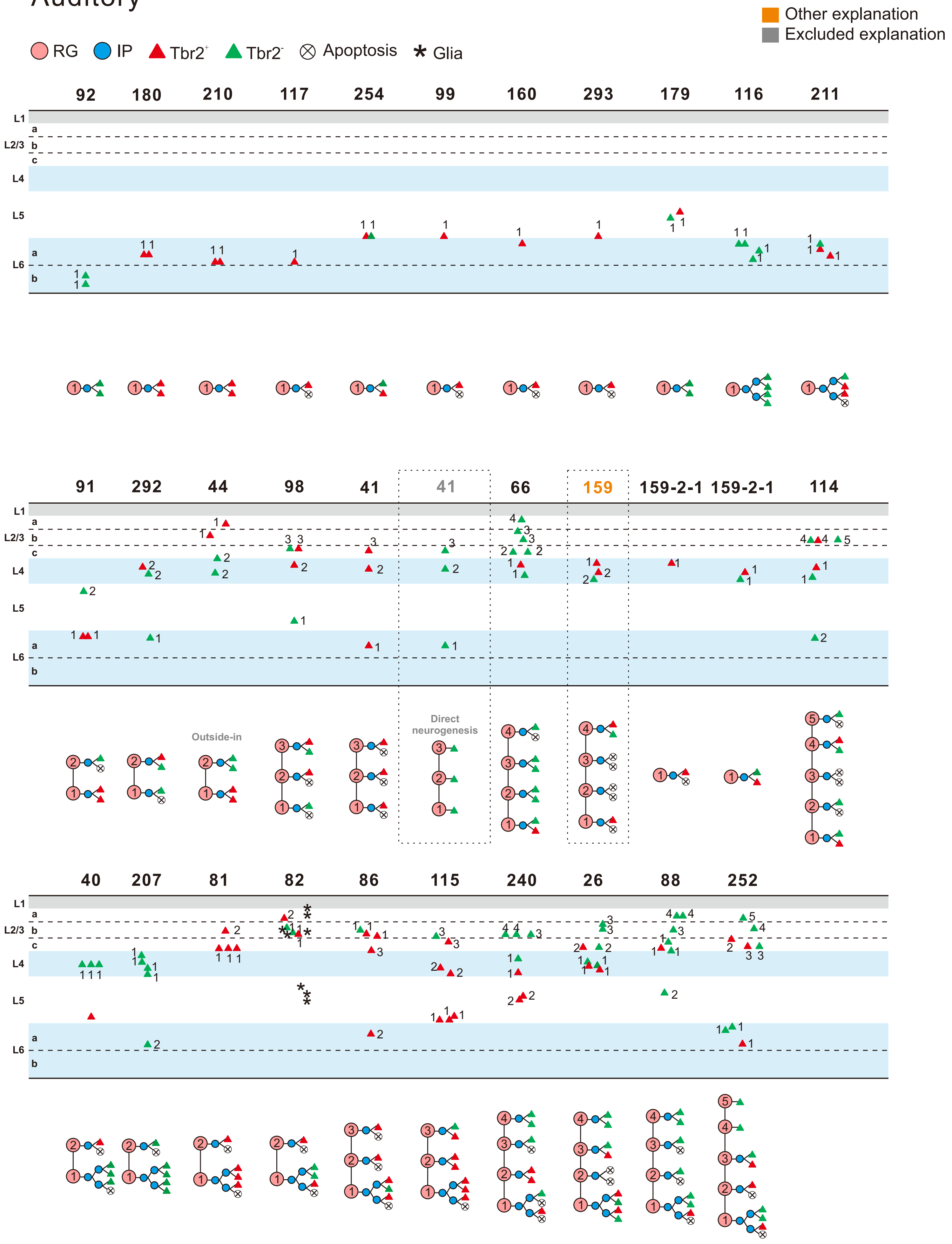

Other explanation
Excluded explanation
RG
IP
Tbr2+
Tbr2-
Apoptosis
Glia
92 180 210 117 254 99 160 293 179 116 211
L1
a
L2/3 b
c
L4
L5
a
L6
b
91 292 44 98 41 41 66 159 159-2-1 159-2-1 114
Outside-in
Direct neurogenesis
40 207 81 82 86 115 240 26 88 252
