# Supplementary material for: The characteristics of excitatory lineage differentiation and the developmental conservation in Reeler neocortex
Source: Cell Prolif. 2023 Dec 12;57(5):e13587. doi: 10.1111/cpr.13587 (PMC11056708; doi:10.1111/cpr.13587)

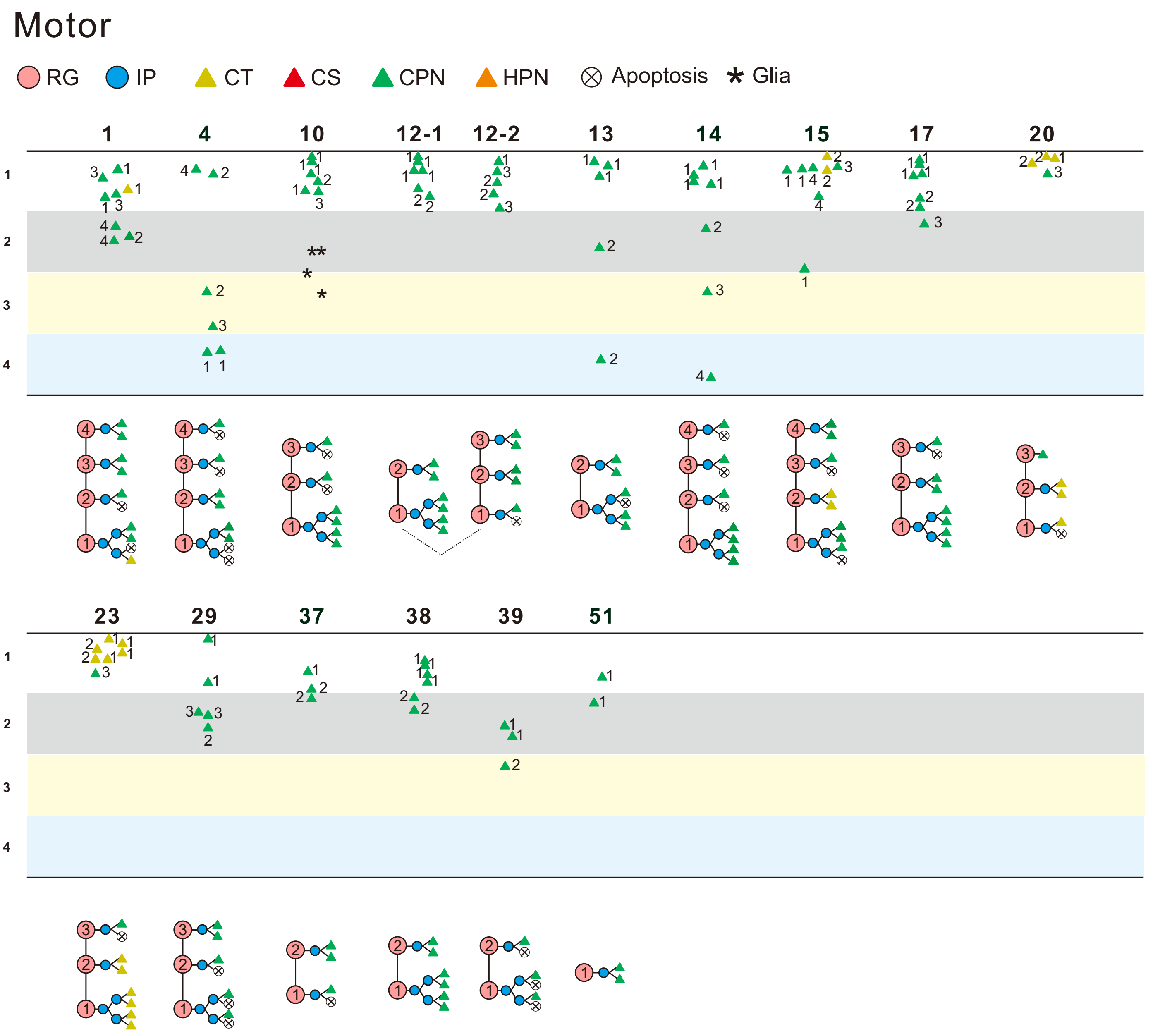
Motor
RG
IP
CT
CS
CPN
HPN
Apoptosis
Glia
1
4
10
12-1
12-2
13
14
15
17
20
23
29
37
38
39
51

Somatosensory

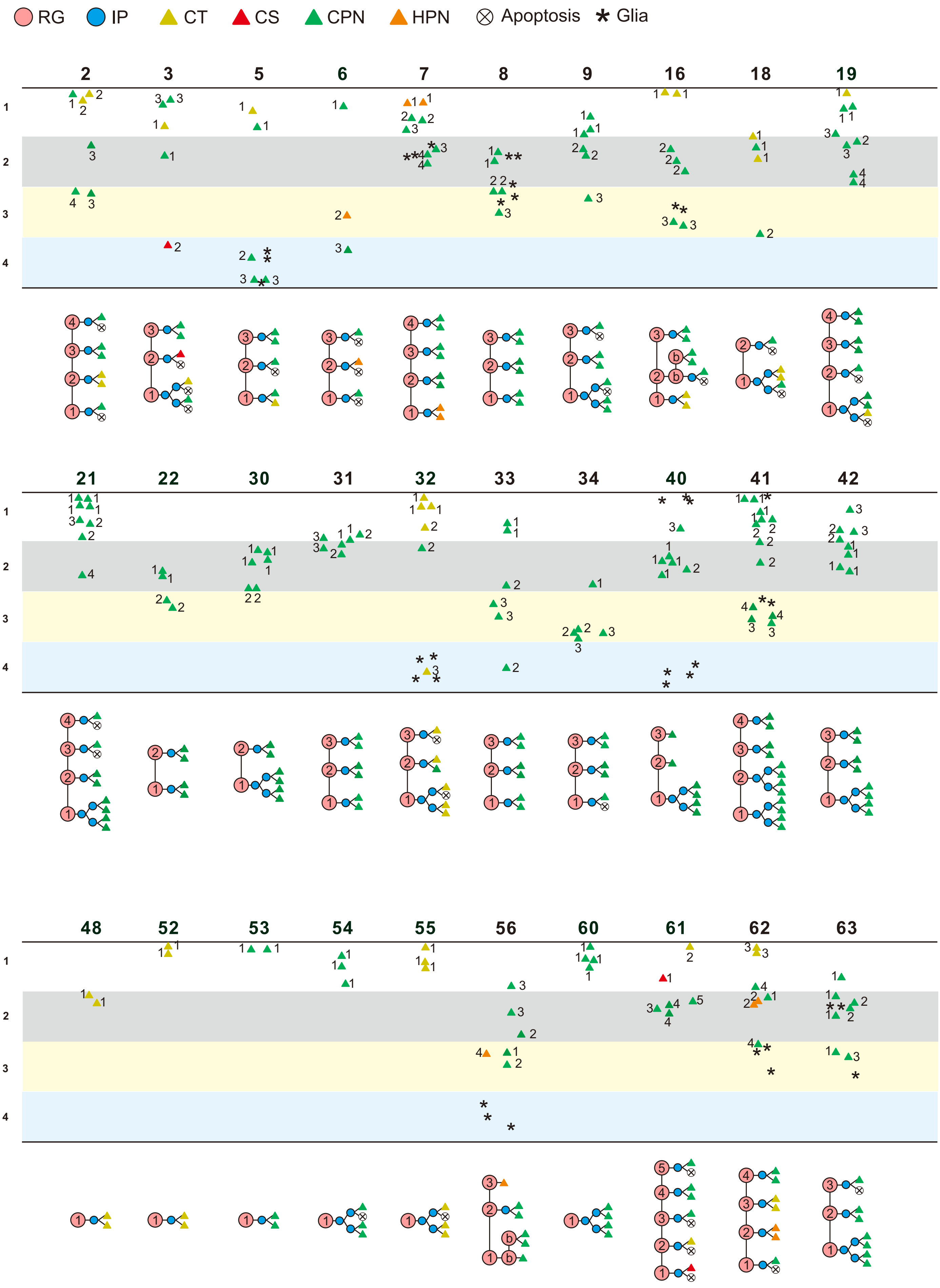

Somatosensory

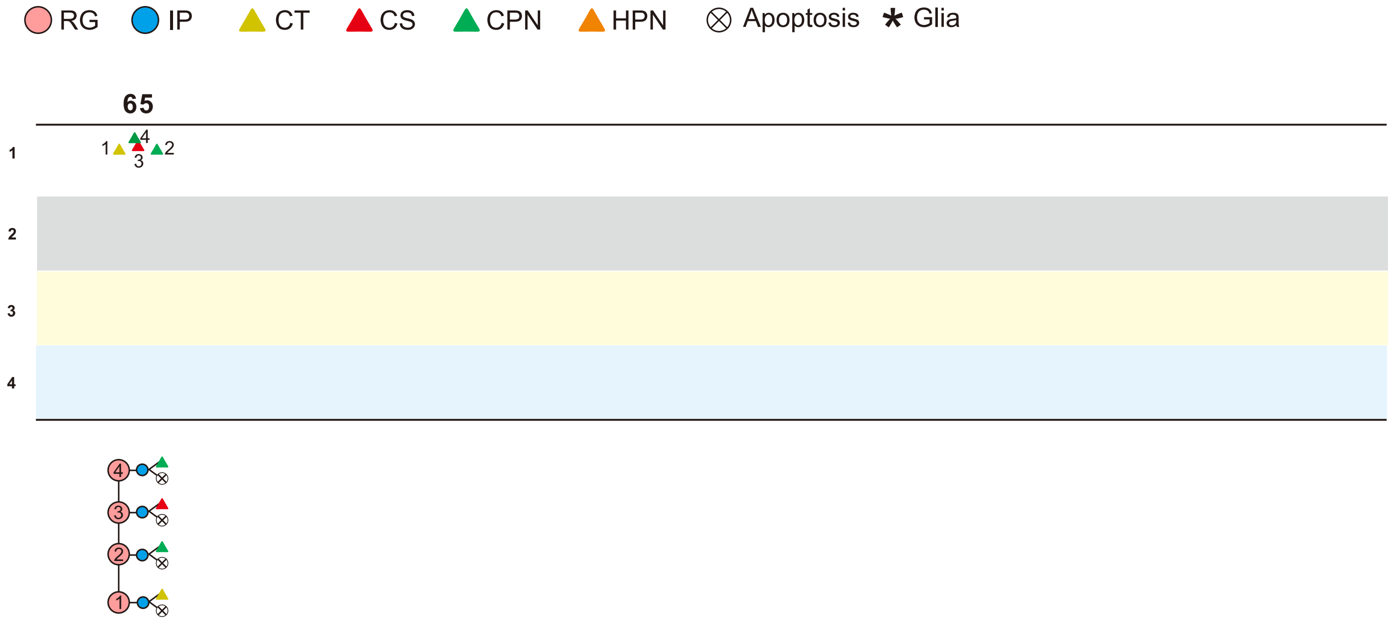

Visual

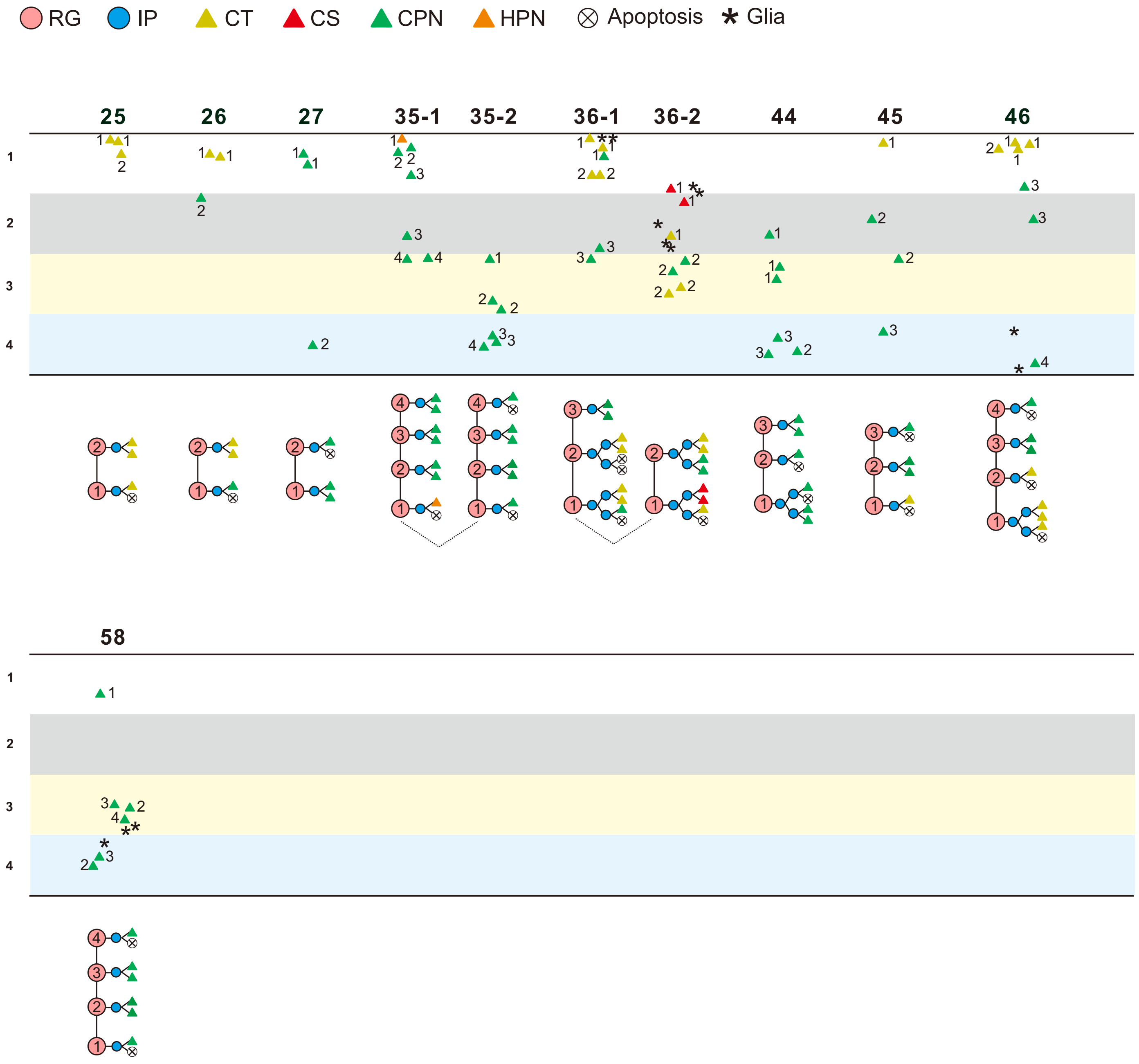
RG
IP
CT
CS
CPN
HPN
Apoptosis
Glia
25
26
27
35-1
35-2
36-1
36-2
44
45
46
58

Auditory

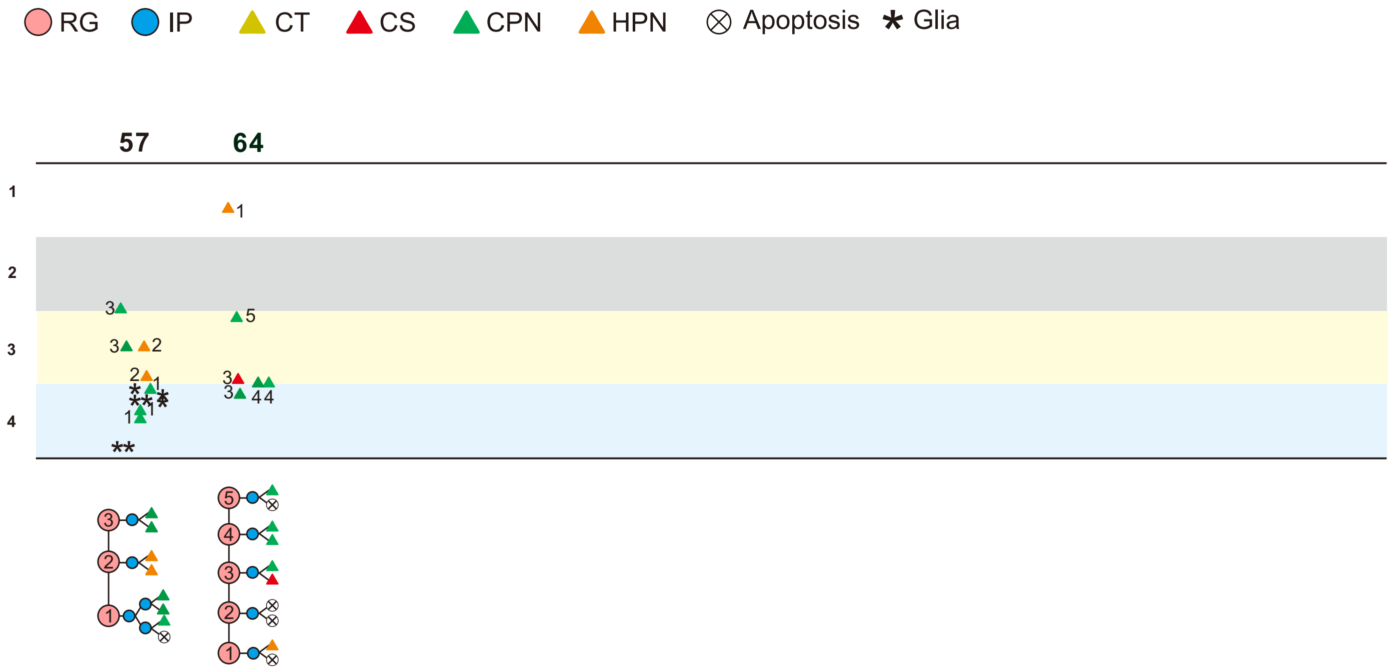

Other

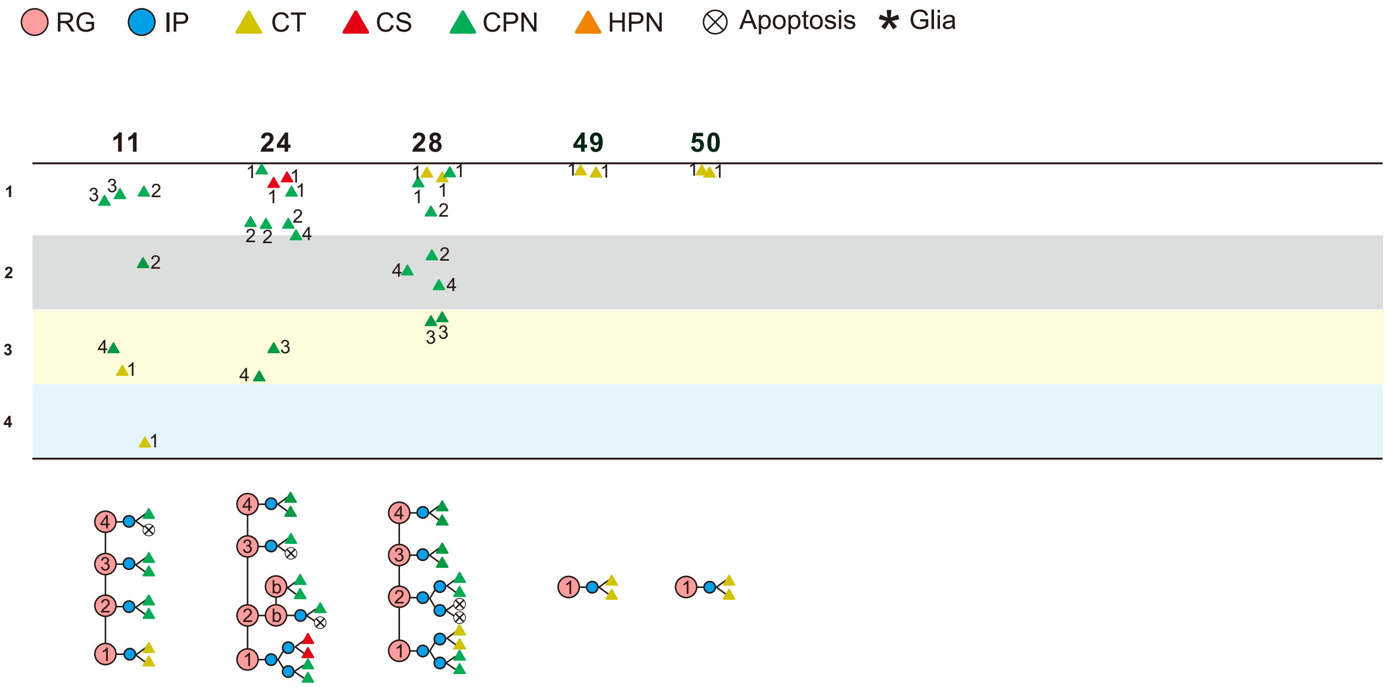

Supplement: Supplementary file 1 — Data S1. Supporting Information. [file CPR-57-e13587-s001.zip › Supplementary diagram 2.pdf]
