# Supplementary material for: The characteristics of excitatory lineage differentiation and the developmental conservation in Reeler neocortex
Source: Cell Prolif. 2023 Dec 12;57(5):e13587. doi: 10.1111/cpr.13587 (PMC11056708; doi:10.1111/cpr.13587)
Supplement: Supplementary file 1 — Data S1. Supporting Information. [file CPR-57-e13587-s001.zip › Supplementary Figure.pdf]

Supplementary Figure 1

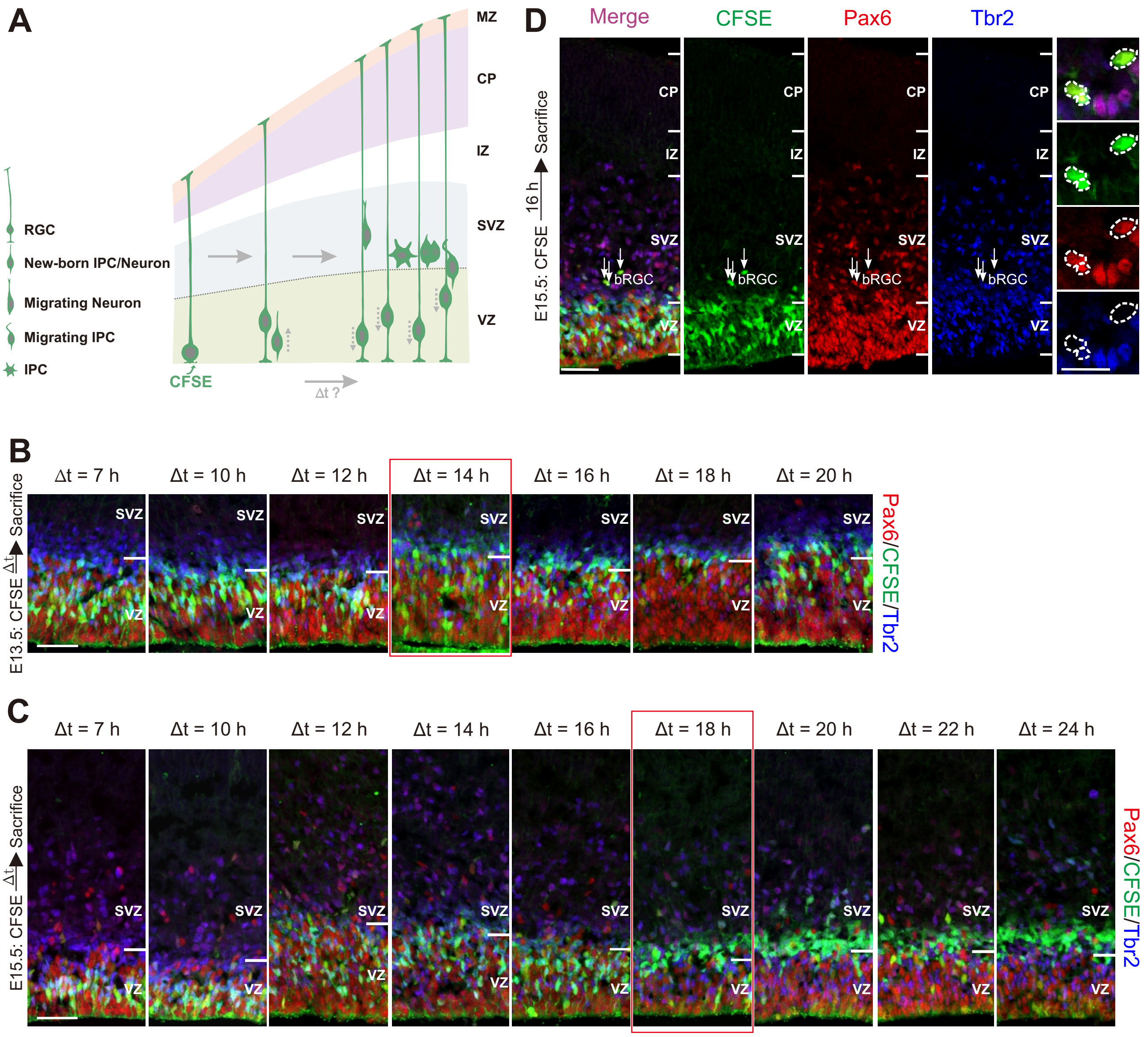

Figure S1: Testing the time window of the CFSE^{+} cells arriving at SVZ

A: Schematic diagram illuminates the strategy of labelling the cells newborn from RGCs by CFSE. B: Testing the time window of newborn cells which start to enter SVZ at E13.5. Triple immunohistochemistry with CFSE (newborn cells, green), Pax6 (RGCs, red) and Tbr2 (IPCs, blue) at E13.5. Scale bar: 50 μm. C: Testing the time window of newborn cells which start to enter SVZ at E15.5. Triple immunohistochemistry with CFSE (newborn cells, green), Pax6 (RGC, red) and Tbr2 (IPC, blue). Scale bar: 50 μm. D: bRGCs are observed at 16 h after ventricular injection at E15.5. Immunostaining with CFSE (newborn cells, green), Pax6 (RGCs, red) and Tbr2 (IPCs, blue). bRGCs are indicated as CFSE^{+}Pax6^{+} Tbr2^{-} and arrows shown bRGCs labelled by CFSE in SVZ. Scale bars: 50 μm (left) and 20 μm (right).

Supplementary Figure 2

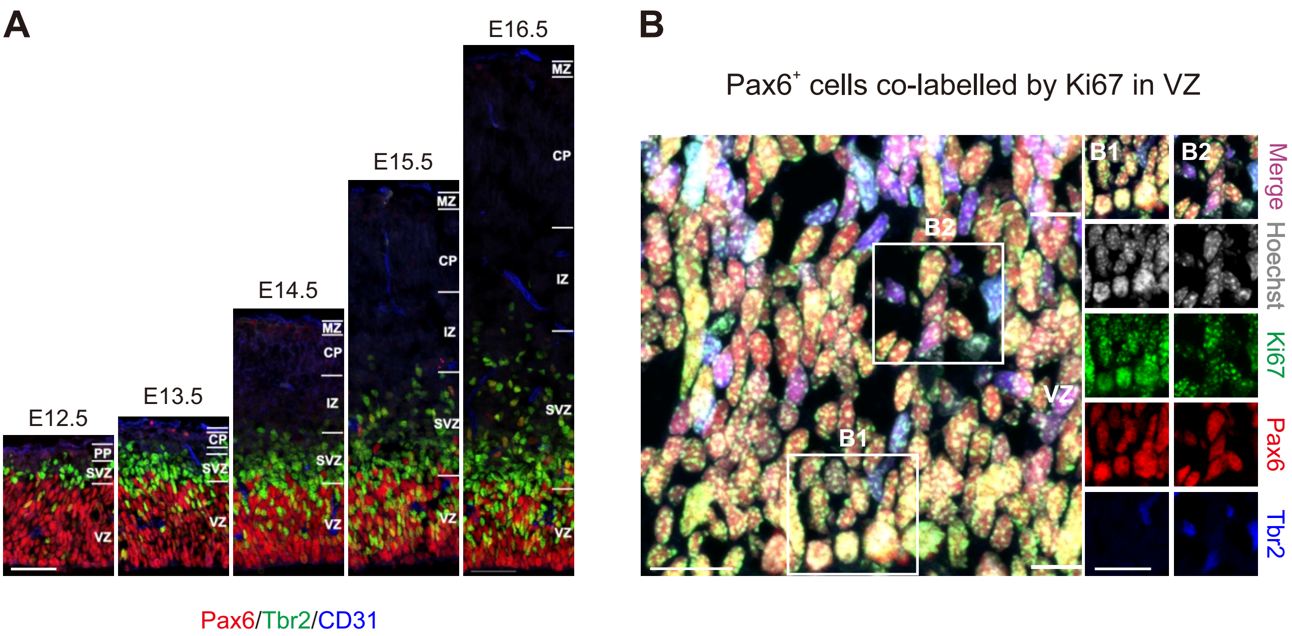

Figure S2: Cell type and their distribution in embryonic VZ

A: Low-magnification images of embryonic neocortex from E12.5 to E16.5 stained with antibodies against Pax6 (red), Tbr2 (green) and CD31 (blue). Scale bar: 50 μm. B: RGCs stained with Ki67 in VZ. Cells expressing Pax6 (red) also express Ki67 (green) at ventricular surface (B1) and under VZ/SVZ boundary (B2). Scale bar: 20 μm.

Supplementary Figure 3

A

E14.5 ──► 16 h

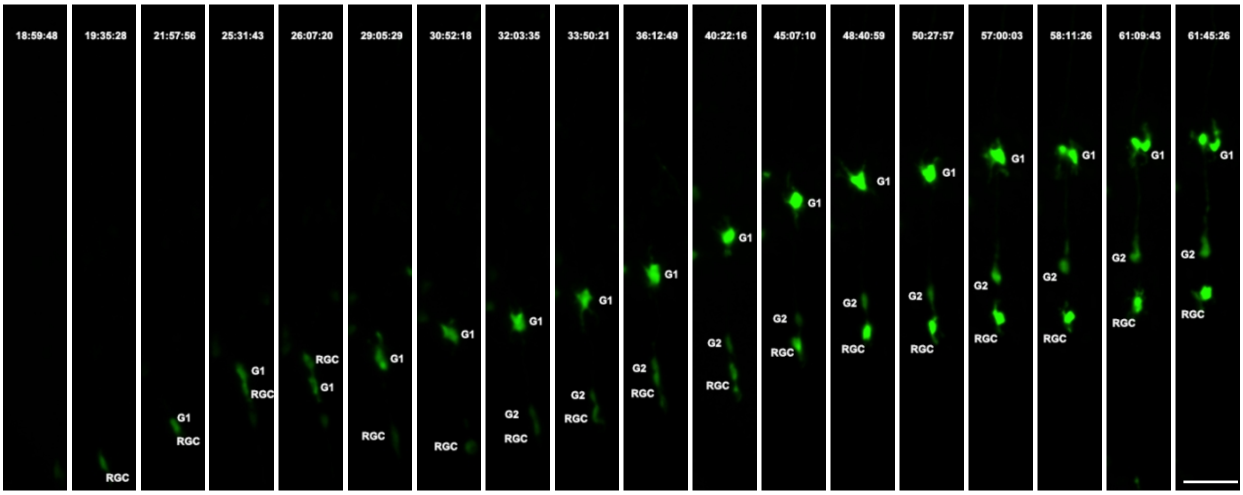

Figure S3：Time-lapse microscopy capture the process of IPC generation and division

A: Time-lapse imaging of a single RGC labelled by retrovirus at E14.5, the slice is collected at 16 h after retrovirus injection. RGC generate the first generation (G1) of IPC that divide symmetrically in the SVZ to generate two daughter cells, while the second generation (G2) of IPC remains under the VZ/SVZ boundary. Scale bar: 50 μm.

Supplementary Figure 4

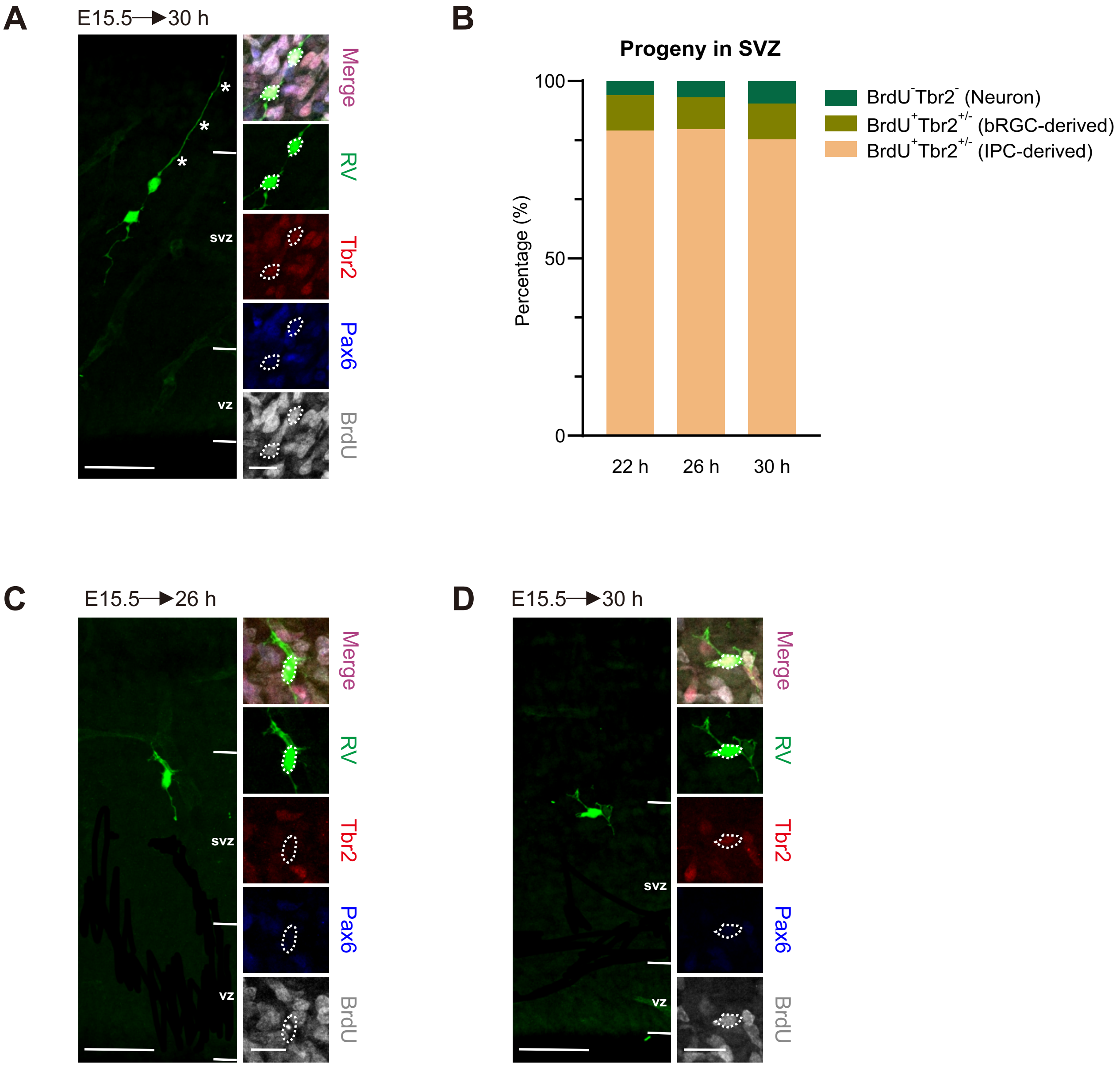

Figure S4: Clones of bRGC, apoptosis and direct neurogenesis labelled by retrovirus at E15.5

A: Representative image of the bRGC division labelled by retrovirus at 30 h after injection, the bRGC has a long basal process but no apical process, the asterisk indicated bRGC basal process. High-magnification images are shown to the right. The cells co-stained with BrdU (white), Tbr2 (red) and Pax6 (blue). Scale bars: 50 μm (left) and 20 μm (right). B: Quantification of the percentage of bRGC clones in the SVZ at E15.5. The clones are collected from different time intervals (22 h, 26 h and 30 h, respectively) after retrovirus injection. C: Representative image of the neuron from direct neurogenesis labelled by retrovirus at 26 h after injection. High-magnification images are shown to the right. The cells co-stained with BrdU(white), Tbr2 (red) and Pax6 (blue). Scale bars: 50 μm (left) and 20 μm (right). D: Representative image of the single-cell clone from IPC labelled by retrovirus at 30 h after injection. High-magnification images are shown to the right. The cells co-stained with BrdU (white), Tbr2 (red) and Pax6 (blue). Scale bars: 50 μm (left) and 20 μm (right).

Supplementary Figure 5

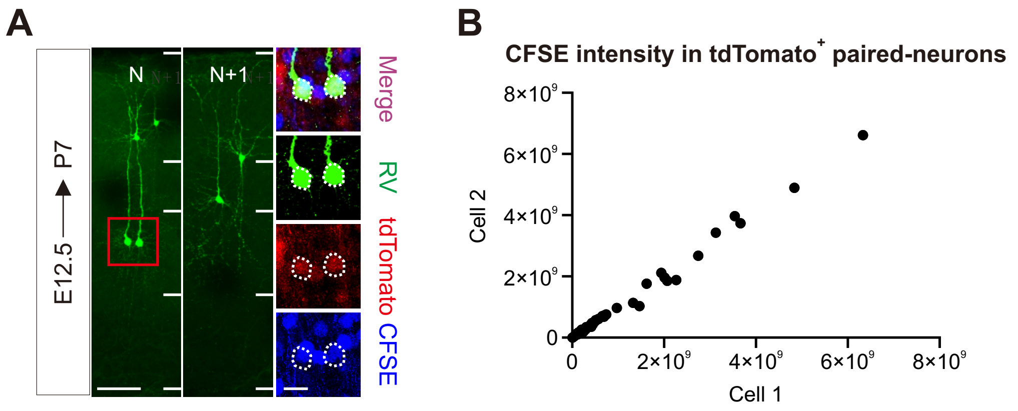

Figure S5: Assessing the accuracy of CFSE that CFSE divide into two progenies equally

A: Paired tdTomato^{+} IPC-deriving neurons induced with tamoxifen from *Tbr2-CreER; Ai14* mice in a lineage. High-magnification images show colocalization of tdTomato (red) with retrovirus (green) and CFSE (blue). We assess the paired tdTomato^{+} IPC-deriving neurons by nearest neighbor distance (NND). Scale bar: 100 µm (left) and 20 µm (right). B: Analysis of whether CFSE distribute into two daughter cells equally. Paired-neurons possess similar intensity of CFSE after division. The intensity of CFSE is measured by Imaris.

Supplementary Figure 6

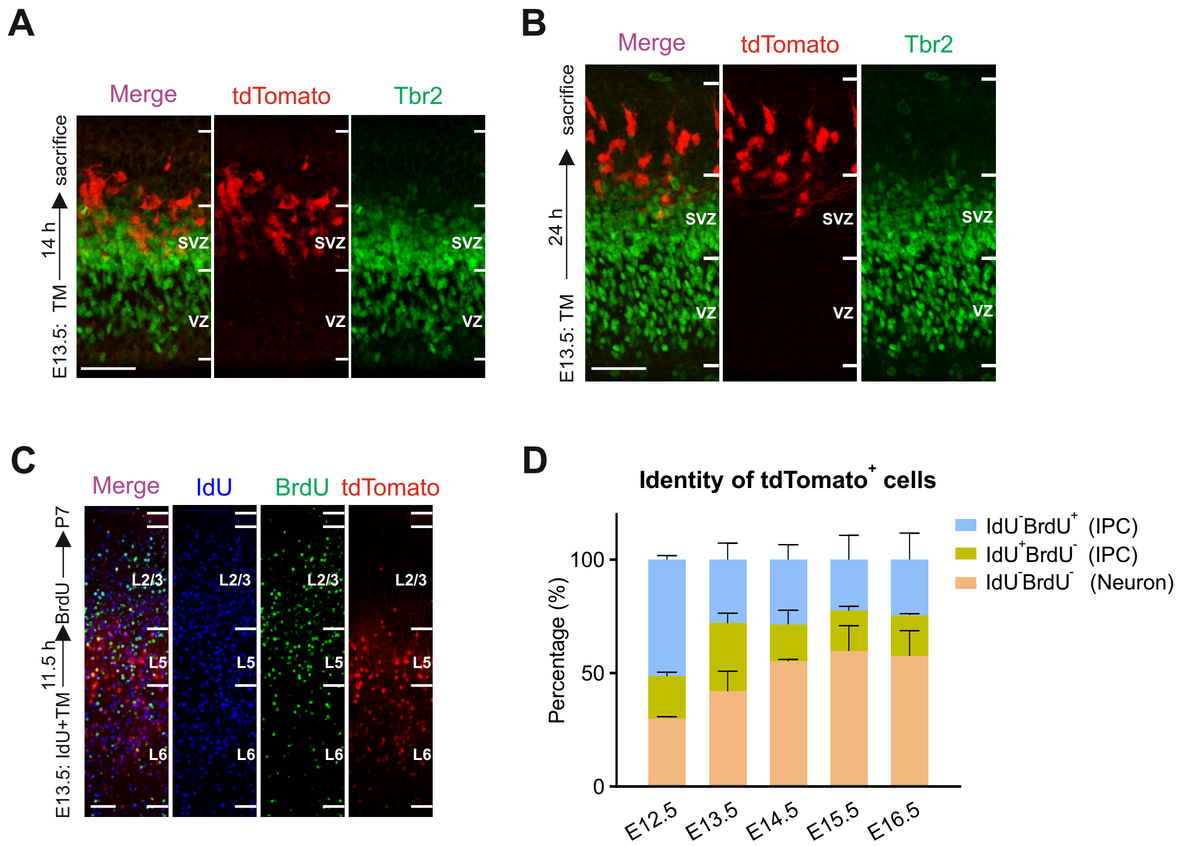

Figure S6: Assessing the cell identity of tdTomato$^+$ cells labelled by *Tbr2-CreER* mice

(A-B): Immunohistochemistry of tdTomato$^+$ cells (red) co-stained against Tbr2 (green). A: None of tdTomato$^+$ cell locate in VZ, part of them have left SVZ at 14 h after tamoxifen (TM) injection. Scale bars: 50 μm. B: Almost all tdTomato$^+$ cells have left SVZ at 24 h after TM injection. Scale bars: 50 μm. (C-D): Cohort of neurons labelled by BrdU after tamoxifen and IdU administration at different embryonic day. C: Representative images show the neocortex at E13.5. Cells are labelled by BrdU (mitotic cell, green) after tamoxifen (tdTomato$^+$ cell, red) and IdU (mitotic cell, blue) administration. Scale bars: 100 μm. D: The percentage of cell identity labelled by *Tbr2-CreER* mice. tdTomato$^+$ cell never co-labeled by IdU or BrdU (IdU$^-$ BrdU$^-$tdTomato$^+$) are considered as neuron, tdTomato$^+$ cell co-labeled by IdU or BrdU (IdU$^+$ BrdU$^-$ or IdU$^-$ BrdU$^+$) are considered as IPC. Quantifications are made from 4 sections from 3 different mice. Data are mean ± SEM.

Supplementary Figure 7

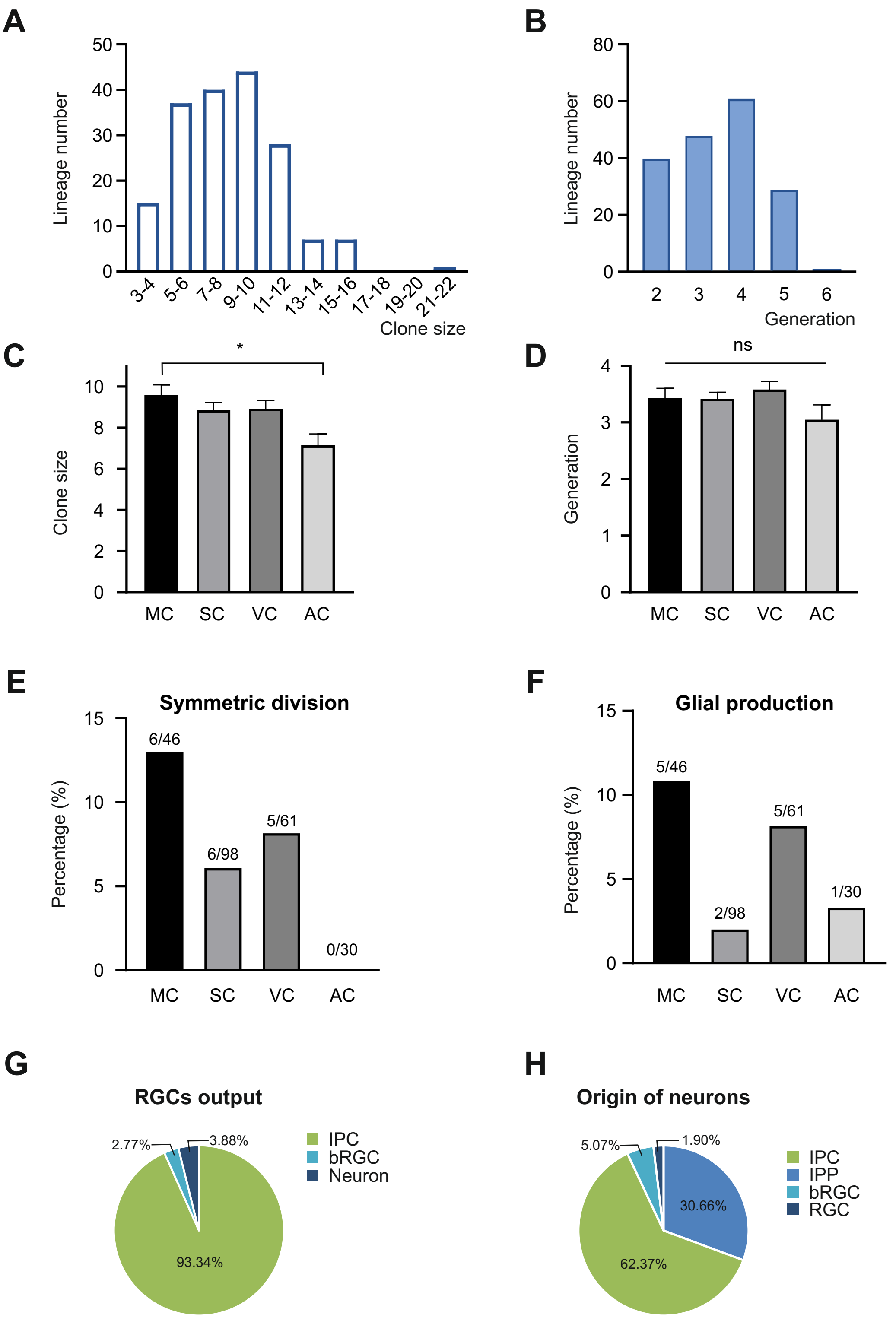

Figure S7: Identities of lineage and pattern of neuron production

A: Analysis of the frequency of lineage with different clone size. B: Analysis of the generation distribution of lineages. C-F: The differences in lineage between brain area. C: The average clone size in four main brain area. D: The average generation in four main brain area. E: The percentage of lineage containing symmetric division in four brain area. F: The percentage of lineage with glial production in four brain area. MC: motor cotex. SC: somatosensory cortex. VC: visual cortex. AC: auditory cortex. G: Percentage of cell types of RGC output in lineage tree, including IPC, bRGC and neuron. H: Percentage of neuronal origin from direct neurogenesis (RGC) and indirect neurogenesis (mediated by IPC, IPP or bRGC).

Supplementary Figure 8

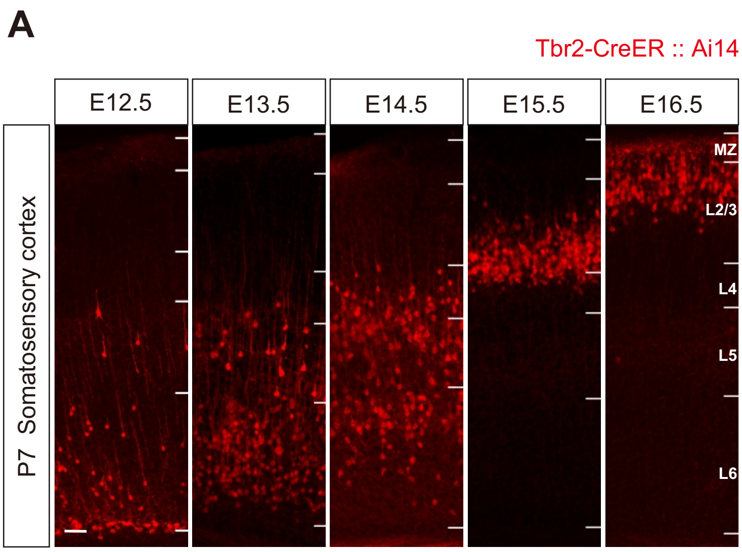

Figure S8: Distribution of tdTomato^{+} cell labelled by *Tbr2-CreER* mice at embryonic stage

A: The distribution of neurons from *Tbr2-CreER; Ai14* mice induced with tamoxifen. Temporal fate mapping by tamoxifen induction from E12.5 to E16.5. Tbr2-CreER mice allow temporary activation to labelling permanent tdTomato observed at P7. Confocal microscopy images depict the distribution within somatosensory cortex. Scale bar: 50 μm.

Supplementary Figure 9

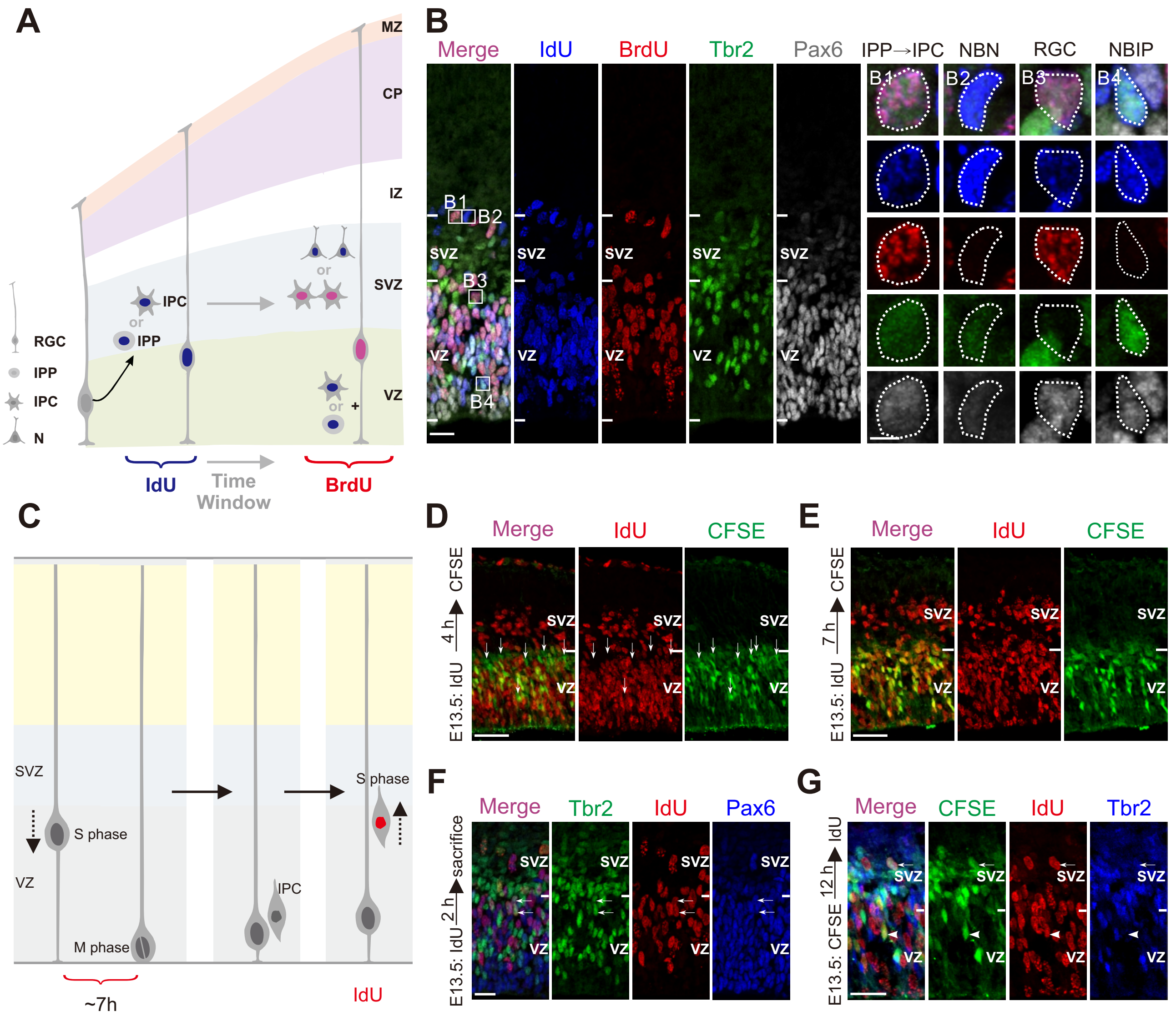

Figure S9: Validation the identity of cells labelled by IdU and BrdU in VZ and SVZ

A: Schematic diagram for distinguishing IPP-derived and IPC-derived cells using IdU and BrdU. B: Immunohistochemistry with the antibodies against IdU (blue), BrdU (red), Tbr2 (green), and Pax6 (white) validate the cell identity in VZ and SVZ. The $IdU^{+}BrdU^{+}$ cells (B1) in SVZ co-labeled with Tbr2 are considered as IPP-derived IPC (divide completely) or IPP (divide incompletely), $IdU^{+}BrdU^{-}$ cells (B2) in SVZ are considered as newborn neuron (NBN), $IdU^{+}BrdU^{+}$ cells (B3) in VZ are indicated as RGCs because they co-labeled with Pax6 only, and $IdU^{+}BrdU^{-}$ cells (B4) in VZ are indicated as newborn IPC (NBIP) because they co-labeled with Pax6 and Tbr2. Scale bar: 20 μm (left) and 5 μm (right). C: Schematic diagram of testing the time window of RGC from S phase to M phase. D: CFSE is injected into ventricular 4 h after IdU administration, arrows indicate the cells only labelled by CFSE (green) without IdU (red). Scale bar: 50 μm. E: CFSE is injected into ventricular 7 h after IdU administration, majority of cells labelled by CFSE are infected by IdU. Scale bar: 50 μm. (F-G): Testing the time point of newborn IPC entering S phase. F: Newborn cells in VZ costained with Tbr2 (green) and Pax6 (blue). Arrows indicate $IdU^{+}Tbr2^{+}$ cells (IPCs) locate in the boundary of VZ/SVZ. Scale bar: 50 μm. G: Detecting the newborn IPCs ($CFSE^{+}Tbr2^{+}$) whether enter into S phase before they arrive at VZ/SVZ boundary. Arrow indicates an $IdU^{+}CFSE^{+}Tbr2^{+}$ cell (IPC) located in the boundary of VZ/SVZ, while none of IdU^{+} IPC located in VZ, arrowhead in VZ indicate an RGC ($IdU^{+}CFSE^{+}Tbr2^{-}$). Scale bar: 50 μm.

Supplementary Figure 10

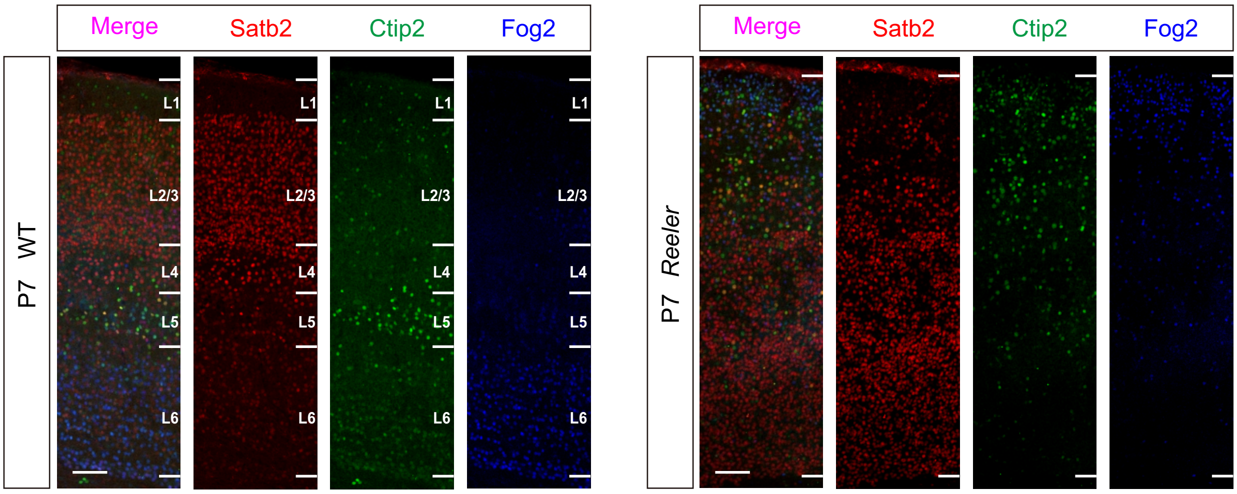

Figure S10: The disordered distribution of neurons in *Reeler* mice

The left images show the normal distribution of neurons distinguished by Satb2 (CPN, red), Ctip2 (SCPN, green) and Fog2 (CThPN, blue). The right images show the disordered distribution in *Reeler* mice. Scale bar: 100 µm.

Supplementary Figure 11

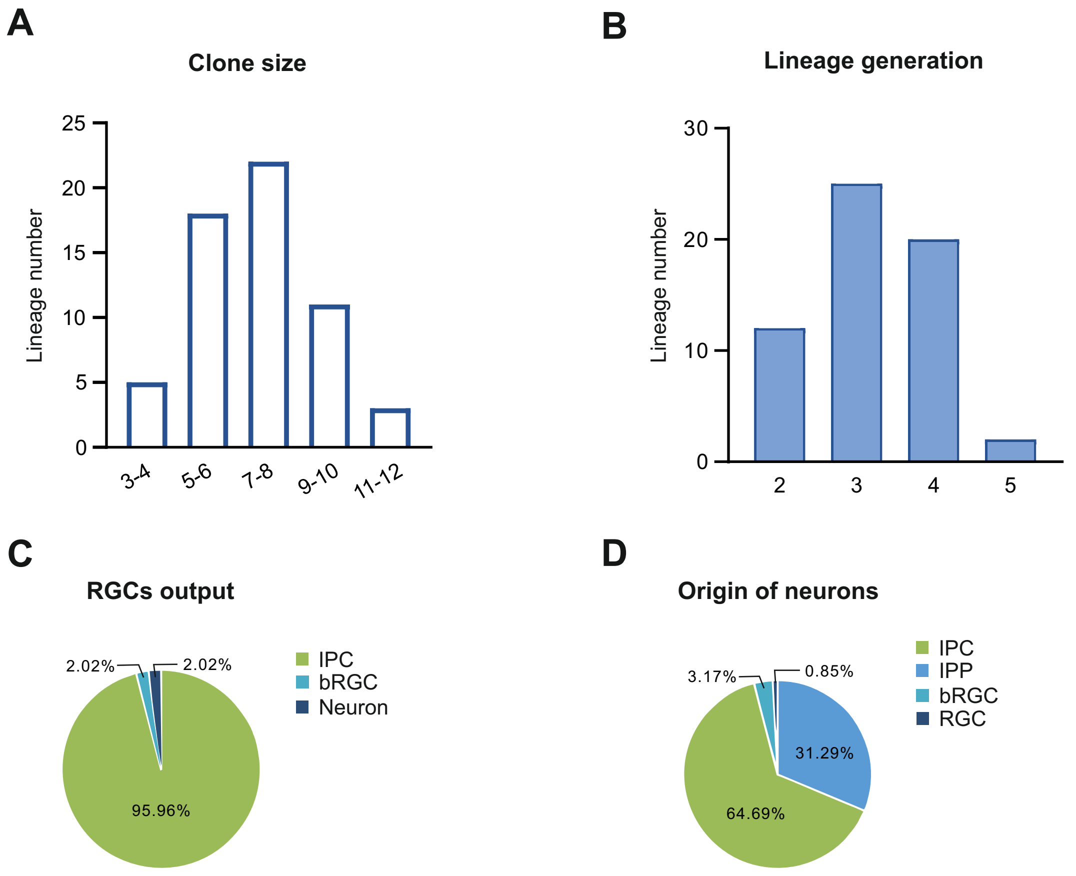

Figure S11: Identities of lineage and pattern of neuron production in *Reeler* mice

A: Analysis of the frequency of lineage with different clone size. B: Analysis of the generation distribution of lineages. C: Percentage of cell types of RGC output in lineage tree, including IPC, bRGC and neuron. D: Percentage of neuronal origin from direct neurogenesis (RGC) and indirect neurogenesis (mediated by IPC, IPP or bRGC).
